# Comparative phenotypic, genotypic and genomic analyses of *Bacillus thuringiensis* associated with foodborne outbreaks in France

**DOI:** 10.1371/journal.pone.0246885

**Published:** 2021-02-19

**Authors:** Mathilde Bonis, Arnaud Felten, Sylvie Pairaud, Angélie Dijoux, Véronique Maladen, Ludovic Mallet, Nicolas Radomski, Arnaud Duboisset, Chantal Arar, Xavier Sarda, Gaelle Vial, Michel-Yves Mistou, Olivier Firmesse, Jacques-Antoine Hennekinne, Sabine Herbin

**Affiliations:** 1 Laboratory for Food Safety, French Agency for Food, Environmental and Occupational Health & Safety (ANSES), Université Paris-Est, Maisons-Alfort, France; 2 Regulated Products Assessment Department, French Agency for Food, Environmental and Occupational Health & Safety (ANSES), Université Paris-Est, Maisons-Alfort, France; Institut National de la Recherche Agronomique, FRANCE

## Abstract

*Bacillus thuringiensis* (Bt) belongs to the *Bacillus cereus* (Bc) group, well known as an etiological agent of foodborne outbreaks (FBOs). Bt distinguishes itself from other Bc by its ability to synthesize insecticidal crystals. However, the search for these crystals is not routinely performed in food safety or clinical investigation, and the actual involvement of Bt in the occurrence of FBOs is not known. In the present study, we reveal that Bt was detected in the context of 49 FBOs declared in France between 2007 and 2017. In 19 of these FBOs, Bt was the only microorganism detected, making it the most likely causal agent. Searching for its putative origin of contamination, we noticed that more than 50% of Bt isolates were collected from dishes containing raw vegetables, in particular tomatoes (48%). Moreover, the genomic characterization of isolates showed that most FBO-associated Bt isolates exhibited a quantified genomic proximity to Bt strains, used as biopesticides, especially those from subspecies *aizawai* and *kurstaki*. Taken together, these results strengthen the hypothesis of an agricultural origin for the Bt contamination and call for further investigations on Bt pesticides.

## Introduction

*Bacillus thuringiensis* (Bt) is a ubiquitous, Gram-positive bacterium, well known for its ability to produce insecticidal crystals during sporulation [1, 2]. These crystals are composed of δ-endotoxins classified within a large family of Cry/Cyt toxins (more than 850 halotypes described so far), firstly according to their specificity of action against insects [3], and secondly according to their sequence similarity [4]. When activated in the midgut of larvae, the toxins create pores in the epithelial membrane leading to gut paralysis and to systemic diffusion of bacterial spores. Insects die of starvation or septicemia, or both [5].

Due to this intrinsic characteristic, Bt is used worldwide in organic and conventional farming (to control *Lepidoptera* and *Coleoptera*), for mosquitoes control, and to protect forests against defoliator insects. Consequently, Bt is the most widely used bacterial biopesticide, with more than 50% of the world sales/market shares in 2010 [6]. Moreover, this trend is likely to accelerate in the next few decades in France since the governmental policies aim to reduce by half the use of chemical phytopharmaceuticals, in favor of biocontrol agents, by 2025. Therefore, exposure via food of non-targeted organisms, including humans, will increase along with the use of Bt products. This raises the question of the real impact of their consumption by humans and the need for efficient monitoring tools.

Bt belongs to the large group of opportunistic bacteria *Bacillus cereus sensu lato* (Bc), whose classification is complex and regularly revisited [7]. This group contains at least eight species, spread across seven phylogenetic clades (I to VII), defined by a part of the *panC* gene sequence, with Bt distributed among four of them (II to VI) [8, 9]. Bc is well-known for its involvement in various infectious diseases in humans, in particular foodborne outbreaks (FBOs) associated with diarrheal or emetic-type symptoms. In 2017, Bc was considered the second most common causative agent of FBOs in France [10], and the third most common in Europe [11], with 298 food poisoning reports from European Union members states. These FBOs involved 3,184 infected people, 189 hospitalizations, and one fatal outcome [11]. Bc can also cause non-intestinal diseases, such as ocular infections [12] or septicemia in elderly or immuno-compromized individuals [13, 14]. In spite of this, there are currently no European food safety criteria for Bc, except in the case of dried preparations intended for children under six months of age [15], whereas an alert threshold of 10^5^ CFU/g of food has been established in France for general foodstuffs [16].

Although the pathogenicity mechanisms of Bc have not been fully elucidated, they appear to involve the synergistic action of several factors, including cell surface proteins and secreted toxins. Among them, the hemolysins (Hly I to IV) are well known for their cytotoxicity [17] and for the necrotic properties, especially in the case of CytK (Hly IV), whose CytK1 variant is highly toxic [18, 19]. Although less powerful than CytK1, CytK2 has also been found to have hemolytic properties and toxicity towards mammalian cells [20]. The tripartite toxins Hbl(C/D/A) and Nhe(A/B/C) have been found to play a decisive role in virulence by inducing hemolysis, vascular permeability, dermonecrosis or inflammation [21, 22]. While the enterotoxins Hbl, CytK and Nhe appear to be responsible for the diarrhoeal-type of food poisoning, the cereulide toxin, whose synthesis relies on the presence of the *ces* locus, has been described for its involvement in vomiting-type symptoms and cellular damages [23].

As a member of the Bc group, Bt can harbor certain virulence genetic markers, such as the enterotoxin genes [24]. For this reason, it has been hypothesized that Bt could be responsible for part of the Bc-associated illnesses [25]. However, its involvement has been poorly described compared to Bc, due to the absence of a routine test to distinguish Bt from others Bc species. Nonetheless, a Canadian study retrospectively analyzed 39 Bc-FBOs in 2008 and found that Bt was present in 28% of outbreaks, and was the only microorganism identified in 10% of them [26]. In 2012, a familial FBO described in Germany was suspected to have been caused by the ingestion of lettuce on which a *Bt*-based *insecticide was identified* [27]. Additionally, Johler *et al*. more recently found that Bt strains isolated from FBOs were indistinguishable from those found in commercially available Bt products (based on genes profiling and cytotoxicity assays), suggesting that Bt used as a bioinsecticide may be associated with a potential hazard for consumers [28]. In view of repeated reports of suspected cases, the European Food Safety Authority finally recommended in 2016 to conduct more thorough investigations on the putative risk that may represent the use of Bt products may involve for human health [27].

The aim of the present study was first to estimate the frequency of Bt isolation in the context of FBOs that occurred in France and second, to assess their genetic diversity and to compare them with Bt strains isolated from commercially available products. Bt isolates were identified from a large collection of FBO-associated Bc isolates from France, based on the detection of parasporal crystals. Phenotypic and genomic characterizations were then conducted to compare isolates both pairwise and against a representative panel of Bt strains from the European market.

## Materials and methods

### FBO-associated isolates and data collection

For this study, we used a total of 1,263 *B*. *cereus sensu lato* (Bc) isolates previously collected in France from food during investigations of 250 French foodborne outbreaks that occurred in the country between 2007 and 2017. Outbreaks were declared to local health authorities, who collected epidemiological data through interviews or questionnaires. They included the date of the FBO, the nature of the consumed dishes, the number of patients, the symptoms, and the time before onset of symptoms. Based on these data, hypotheses about the etiological agent were established and food samples were collected for analysis. The detection and the counting of Bc isolates from food were performed by certified French laboratories, as previously described [29], and according to the International Organization for Standardization (ISO) 7932 standard method. Between 1 and 15 colonies per FBO or foodstuff were selected and transferred to the “*Staphylococcus*, *Bacillus* and *Clostrium”* unit of ANSES for further analysis and characterization. Information related to the putative detection of other food microorganisms was also collected.

### Identification of Bt isolates within the Bc collection

The FBO-associated Bc collection was screened for the presence of Bt isolates. As no standardized procedure was available to distinguish Bt from other Bc, we used a method consensually accepted at the international standardization level (ISO/TC34/SC9/WG20), based on the microscopic detection of parasporal insecticide crystals, as follows. Bc isolates were cultivated overnight on tryptone soya agar yeast extract (TSAYE) agar plates (Biokar) at 30°C, then on the solid sporulating medium hydrolysate of casein tryptone (HCT), supplemented with 0.3% glucose, at 30°C [30]. A sample of the culture was then resuspended in 10 μL of distilled water and screened by phase-contrast microscopy every 24h until 72h of incubation. Images were acquired with an Olympus BX51, using the Archimed software. When crystals were observed, corresponding Bc isolates were assigned to the Bt species.

### Examination of incriminated foodstuffs

To investigate the origin of contamination for the Bt isolates, we examined the nature of the food products from which they were isolated, comparatively to Bc (non-Bt) isolates. Since the Bc collection exhibited a clonal redundancy per FBO, 59 Bt and 437 non-Bt representative isolates were selected, based on their original phenotypic and genotypic profiles (see respectively parts “Phenotypic characterization of Bt isolates” and Genotypic characterization of Bt isolates) per FBO and per foodstuff. Then we calculated the frequency of Bt/non-Bt isolates association with eight types of foodstuff: tomatoes (including cooked tomatoes but excluding tomato sauce), lettuce, raw vegetables, fruits (raw and cooked), starch products, meat, fish or seafood and spices or dried herbs.

### Collection and isolation of insecticide Bt isolates

A panel of 19 phytopharmaceutical and biocides products containing single, non-genetically modified Bt strains was collected (Table 2). These preparations were selected to cover all the biopesticide uses of Bt authorized in France under Regulations (EC) No 1107/2009 and No 528/2012. In agreement with the labeling satisfying the requirements laid down in the Regulation (EC) No 1107/2009, they included altogether 10 different Bt strains, divided into 4 subspecies: *aizawai*, *kurstaki*, *israelensis* and *morrisoni*. For isolation, one g of each product was resuspended in 9 mL of triptic soy broth (TS) until complete dissolution, and bacteria were isolated by successive plating on selective media (Mossel, Biomérieux).

### Phenotypic characterization of Bt isolates

The lecithinase and hemolytic activities of FBO and insecticide Bt isolates were assessed according to the ISO 7932 standard method, as follows. The presence or absence of lecithinase activity was detected by bacterial cultivation on Mossel agar plates (Biomérieux) for 18 to 24 hours (h) at 30°C. When expressing the lecithinase enzyme, isolates exhibited an opaque halo of precipitation surrounding the colonies due to the presence of an egg yolk emulsion. Hemolytic activity was evaluated after inoculating Columbia medium agar plates (Biomérieux) containing 5% sheep blood, with single colonies from TSAYE agar plates 24h-cultures. Plates were incubated for 18 to 24 hours at 30°C before assessing the presence of the hemolytic halo.

### DNA isolation for genotypic characterization

The DNA of Bt isolates was extracted after overnight cultures at 30°C on TSAYE agar plates, using a DNeasy Blood and Tissue Kit (Qiagen). Briefly, a few colonies were resuspended in 180 μL of 1M Tris-HCl, 0.25M Sodium-EDTA, 1.2% TritonX-100 and 2% lysozyme, and incubated for 1 hour at 37°C. The following steps comply with the manufacturer’s recommendations. DNA was quantified by absorbance measurement at 260 nm, using the NanoDrop1000 spectrophotometer (Thermo Fisher Scientific).

### Genotypic characterization of Bt isolates

Bt isolates were genotypically characterized according to the method detailed in S1 Table. The presence of the *cytK1/2*, *ces*, *hlyII*, *nheA/B/C* and *hblA/C/*D genes was determined by PCR with the ProFlex PCR System (Applied Biosystems), using respective primers and methods (S1 Table). To assign Bt isolates to one of the seven described phylogenetic groups, a part of the *panC* gene was amplified as described (S1 Table) and sequenced (Eurofins MWG Operon). For publicly available sequences, the full sequence of *panC* was extracted with BLAST [31], using the ATCC 14579 *panC* sequence (AE016877.1) as the query. The phylogenetic classification was then performed online (https://www.tools.symprevius.org/Bcereus/) using a public algorithm based on *panC* sequence similarity and the statistical significance of matches with database sequences [9]. All the PCR products were analyzed by electrophoresis onto 2% agarose gels (made with a 50/50 mix of Seakem® GTG™ Agarose and NuSiev® GTG™ Agarose, Lonza), with a run in 1X TBE, for 1.5 hours at 90V.

### M13-PCR typing

To study the diversity of Bc isolates, we used a coliphage M13 sequence-based PCR (M13-PCR) derived from a Random Amplified Polymorphic DNA (RAPD) technique and adapted from [32], according to the method described in S1 Table. The PCR products were separated by electrophoresis onto 1% agarose gels (made with a 50/50 mix of Seakem® GTG™ Agarose and NuSiev® GTG™ Agarose, Lonza), with a run in 1X TBE, for 10 min at 50V, followed by 3.5h at 90V. Gels were stained with ethidium bromide. For this study, the electrophoretic patterns of 59 representative FBO-Bt and 19 representative insecticide Bt isolates were analyzed and compared using Bionumerics 7.6. A dendrogram was constructed based on pairwise Dice similarity coefficients calculations [33] and UPGMA clustering, with tolerance and optimisation set at 1%. The Bt isolates were tested three times by M13-PCR typing and similar visual patterns were observed.

### Detection of the Nhe and Hbl enterotoxins

To confirm the *in vitro* expression of *nhe* and *hbl*, the toxins production was assessed from a panel of 10 representative commercial Bt isolates and 21 representative Bt isolates, selected for their original phenotypic and genotypic profiles per foodstuff and per FBO from group A (i.e. for which no other microorganism could be detected). Each Bt isolate was cultivated on TSAYE agar plates for 18 hours at 30°C, then in 10 mL of brain heart infusion broth (BHI, Biomérieux) inoculated with a single colony, for 6 hours at 37°C with stirring. Production of the Nhe and Hbl enterotoxins was then estimated with two immunological tests, using the BDE VIATM kit (3M-Tecra) and the BCET-RPLA kit (Oxoïd), respectively, as previously described [34]. The value indices obtained for FBO-Bt and commercial Bt were compared using the non-parametric Wilcoxon statistical test.

### Whole genome sequencing (WGS)

The DNA of Bt/Bc isolates was extracted using the KingFisher Cell and Tissue DNA kit (ThermoFisher), as follows. Bacteria were grown overnight at 30°C, successively on TSAYE agar plates and then in 10 mL of BHI. Cells from 2 mL aliquots were lysed in 600 μL of 40 mM EDTA, 2 mg/mL lysozyme for 1 hour at 37°C, before being resuspended in 200 μL of lysis buffer and 25 μL of proteinase K, and incubated for 15 minutes at 70°C then 30 minutes at 80°C. RNA was removed by incubating for 5 minutes at 37°C with 12 μg/mL of RNAse A (Promega). The following purification steps were performed using the KingFisher Duo Prime system, according to the manufacturer’s recommendations. Final elution was performed in 100 μL of PCR grade water. To estimate the DNA purity, we measured the absorbance of DNA at 230, 260 and 280 nm using the NanoDrop2000 (ThermoScientific). The ratio A260/A280 and A260/A230 ratios were expected to be comprised between 1.5 and 2.5. The concentration of the solutions was measured with the Qubit Fluorometer (Invitrogen) and the dsDNA HS Assay kit (Invitrogen), according to the manufacturer’s recommendations. The integrity of genomic DNA was checked by visualization onto 0.8% agarose gels (made with a 50/50 mix of Seakem® GTG™ Agarose and NuSiev® GTG™ Agarose, Lonza), with a run in 1X TBE, for approximately 2 hours at 90V. Isolated DNA was sent to the ICM (« Institut du Cerveau et de la Moëlle épinière») for paired-end read sequencing (2x150bp), using the Nextera XT DNA Library Prep Kit and the Nextseq500 sequencing system (Illumina).

### Genome assembly

The reads were assembled with the in-house workflow ARtWORK v1.0 [35], with default parameters. After read decompressing, short paired-end reads were mapped against the closely related reference genome identified by estimating the Jaccard index with Mash v2.0 [36], among a selected collection of high-quality fully closed assemblies, representative of the species (S4 Table). The samples presenting a depth of coverage against the reference lower than 50X were excluded, those between 50X and 100X were retained, and those higher than 100X were normalized at 100X with Bbnorm v38.22 (https://jgi.doe.gov/data-and-tools/bbtools/). Reads were trimmed with Trimmomatic v0.38 (i.e. adapters sequences, quality phred score of 30 and minimum length of 50 bp) and assembled using SPAdes v3.13.0, with the careful option to reduce the number of mismatches and short indels and the automatic k-mer sizes selection [37]. The closest reference genome was used to perform reference-based scaffolding with MeDuSa v1.6 [18, 38] and gap filling with GMcloser v1.5 [39]. Scaffolds smaller than 150 bp were removed from the final assembly.

### k-mer phylogeny

A phylogenetic tree of 234 Bc/Bt genome assemblies (listed in S4 Table) was built from the number of shared k-mers (kmer size of 17 and sketch size of 10,000) with the in-house script QuickPhylo v1.0 (https://github.com/afelten-Anses/QuickPhylo). This tool used Mash to compute a distance matrix based on the Jaccard index and cluster sequences with the neighbor-joining (NJ) method. The midpoint approach was used to root the tree and its visualization was obtained with iTOL v5 [40]. Sixty-two publicly available genome sequences were added from NCBI to enhance the genetic diversity of the dataset.

### Single nucleotide polymorphism (SNP) analysis

To estimate the genetic distance separating FBO and insecticide isolates, we conducted an SNP analysis using the iVARCall2 v1.0 workflow [41] from sequence reads of 154 Bc isolates belonging to phylogenetic group IV (listed in S4 Table). These samples comprised 19 insecticide Bt isolates and 135 FBO isolates (123 Bt and 12 non-Bt). The closed genome of Bt strain HD73 was used as a reference for read mapping (accession NC_020238.1). The IVARCall2 workflow generated a matrix of pairwise SNP distances for each pair and pseudogenome sequences. These pseudogenomes were reconstructed by replacing the genotypes of detected variants into the reference genome for each paired-end reads of the dataset. Recombination events were excluded to infer the presented phylogenomic reconstruction using ClonalFrameML v1.12, run with 100 simulations [42]. A phylogenetic reconstruction was performed from pseudogenomes using IQ-TREE v1.6.9 [43]. The K3P model was selected as the best fitting model of reconstruction and 10,000 ultrafast bootstraps were generated. Visualization of trees was obtained with iTOL v5 [40].

### Comparison of pairwise SNP distances

The comparison of pairwise SNP distances was performed from the matrix generated by iVARCall2 [41], using an R script adapted from a public script (https://github.com/andersgs/harrietr). According to the phylogenetic reconstruction and calculated SNP distances, each isolate was attributed to one of the 5 defined clusters (hereafter referenced from “a” to “e”). Then, intra- and inter-clusters SNP distances were computed and compared with a Wilcoxon statistical test. Their distribution was illustrated with box plot diagrams.

### Accessory genome analysis

As an addition to the SNP analysis approach, the accessory genomes of the 154 FBO and insecticide isolates were compared. A tree was built using ROARY v3.12.0 with default parameters [44] and iTOL v5 [40]. Similarly to the comparison of pairwise SNP distances, the intra- and inter-clusters pairwise numbers of different genes were computed and compared.

## Results

### Distinction of *Bacillus thuringiensis* (Bt) among FBO-associated Bc isolates

A collection of 1,263 Bc isolates associated with 250 distinct FBOs was established and characterized between 2007 and 2017. Retrospectively, we tested the ability of these isolates to produce crystals under sporulating conditions by phase-contrast microscopy, in order to identify the putative presence of Bt isolates. Similarly to the positive control CIP53137, Bt isolates exhibited the production of intracellular inclusions after 48 h of incubation, followed by the release of crystals and spores (S1 Fig). By contrast, the Bc strain ATCC14579, used as a negative control, was unable to produce crystals. Hence, we identified 143 Bt isolates, representing 11.3% of the total collection of 1,263 Bc. More importantly, these 143 Bt isolates were associated with 49 toxic episodes (listed in Table 1), representing 19.6% of the 250 FBOs considered in the present study.

**Table 1:** List of the 49 *Bacillus thuringiensis*-associated foodborne outbreaks in France from 2007 to 2017.

FBO	Bt isolate	Year ^a^	Incriminated food ^a^	Number of patients ^a^	Symptoms ^a^	Incubation period (h) ^a^	CFU/g ^b^
Group A *: Detection of Bt alone							
2	08CEB037 to 38	2008	Rice salad	13	A, N,V	4 to 24	2,00E+03
5	08CEB121 to 123	2008	Tabbouleh	NC	NC	NC	5,00E+03
	08CEB124 to 127		Mixed beef, onion, tomatoes				1,30E+03
8	08CEB145	2008	Raw vegetables	2	A, V	NC	1,90E+03
10	10CEB01 to 05	2010	Lettuce	44	A, V, D	NC	1,00E+03
11	10CEB46 to 51	2010	Tabbouleh	11	A, O	NC	NC
14	14SBCL08	2011	Raw carrots	3	A, D, V	5	5,80E+03
15	14SBCL16	2011	Raw tomatoes	3	N, V, D	3 to 4	5,50E+03
	14SBCL18		Mixed dish with fish				1,10E+03
16	14SBCL20	2011	Raw vegetables, mozzarella	3	N, V, D	2	2,00E+03
17	14SBCL22	2011	Tomatoes, zucchini, corn	9	A, V	8 to 10	4,00E+03
19	14SBCL176 to 180	2012	Mixed vegetables	14	N, V, D	1	<400
20	14SBCL262 to 266	2012	Strawberry cake	2	N	NC	2,00E+03
23	14SBCL370 and 374	2013	Tomatoes, mixed salad	6	A	7	2,20E+02
	14SBCL371 to 373	2013					3,30E+02
34	16SBCL417 to 418	2015	Fresh apple	15	A, V, D	6	<400
35	16SBCL440	2015	Tomatoes, cucumber	2	NC	NC	<400
38	16SBCL1310	2016	Cucumber	50	D, V, O	>24h	<400
43	17SBCL334	2017	Fresh pineapple	9	V, N, A	2	<400
45	17SBCL527 to 528	2017	Pasta salad	140	V	2	1,60E+03
	17SBCL529 to 531						2,40E+03
46	17SBCL619 to 623	2017	Tomatoes, anchovy	2	A	1	7,00E+02
48	17SBCL967 to 971	2017	Tomatoes, mixed salad	5	D, V, N, O	0,3	5,00E+02
Group B *: Detection of Bt in the presence of other putative pathogens							
1	07CEB29 to 32	2007	Raw vegetables	2	A, D, N	NC	1,60E+03
3	08CEB074	2008	Smoked salmon	11	A, D, F	12	3,00E+03
4	08CEB089BAC	2008	Cooked rice	2	NC	NC	1,70E+03
6	08CEB128	2008	Raw vegetables	19	N, V, D	NC	6,00E+02
7	08CEB135 to 136	2008	Chorizo, celery	60	V, D	NC	9,00E+02
	08CEB137 to 139		Raw tomatoes				1,20E+03
9	09CEB68 to 69	2009	Peas	7	A, N, D	4 to 5	2,00E+07
12	11CEB48 to 52	2011	Raw vegetables, blue cheese	9	N, V, D	5 to 8	4,00E+03
13	12CEB17	2012	Frozen paella	2	V, D, O	1	<400
18	14SBCL49	2012	Mixed salad	8	V, F, A	6 to 34	<400
21	14SBCL309 to 313	2013	Cooked lamb	2	A, D, V	4 to 5	3,60E+03
22	14SBCL361	2013	Mimolette	50	V, D	8	<400
	14SBCL362		Beetroot				5,50E+02
	14SBCL364		Tomatoes				<400
24	14SBCL388	2013	Cooked rice	2	D, A	10	>200
25	15SBCL93 to 95	2015	Pineapple	15	D, V, N, A	2	<400
26	15SBCL482	2015	Tomatoes, mozzarella	11	V, D	NC	<400
27	15SBCL598 to 602	2015	Wheat salad	34	D, V, N, A	12	1,80E+03
	15SBCL603 to 607		Tomatoes, mozzarella				2,90E+03
28	15SBCL915	2014	Mixed salad	9	D, V, A	15	>1000
29	15SBCL1007 to 1011	2015	Mixed salad	3	V, D, F	1 to 3	7,00E+05
30	15SBCL1331	2015	Cooked rice	2	N, A, V	1,5	<400
31	16SBCL350	2015	Tomatoes, mozzarella	11	V, D	NC	<400
32	16SBCL372	2015	Tartiflette	4	V, D, F, O	7 to 9	4,20E+06
33	16SBCL379 to 380	2015	Fresh pineapple	4	N, V, O	NC	4,00E+03
	16SBCL381						2,00E+03
36	16SBCL670	2016	Cooked veal	NC	NC	NC	<400
	16SBCL898		Tomatoes, feta				<400
37	16SBCL1122	2016	Mixed salad	NC	D, V	NC	<400
39	16SBCL1549 and 1551	2016	Strawberry	NC	NC	NC	NC
40	16SBCL1643	2016	Salmon pudding	14	V, A	14,5	<400
41	17SBCL01	2016	Salmon dumpling	12	D, V, A	1	<400
42	17SBCL263 to 268	2017	Curry chicken	2	D, F, A	0,25	1,00E+03
44	17SBCL429	2017	Pear crumble	7	V, D	NC	<400
	17SBCL430 to 431		Endive salad				<400
47	17SBCL885 to 889	2017	Stuffed tomatoes	34	V	6	6,00E+01
49	17SBCL1202	2017	Raw vegetables	4	D, A	NC	<400

Abbreviations: FBO = Foodborne outbreak, CFU = colony-forming unit, A = abdominal pain, D = diarrhea, N = nausea, V = vomiting, F = fever, O = other, and NC = not communicated.

^a^ Data were collected during FBO investigations by local health authorities, through interviews.

^b^ The detection and counting of Bc isolates from food were performed by certified laboratories, as previously described [29], and according to the International Organization for Standardization (ISO) 7932 standard method.

* FBOs were classified into 2 groups (A and B) according to the detection of other putative food pathogens during investigation of the same FBO. The search for other pathogens was conducted by certified laboratories, based on clinical data and hypotheses of etiological agents.

### Other food pathogens in Bt-associated FBOs

To determine whether Bt isolates were the putative etiological agents of the 49 FBOs, we examined the potential co-occurrence of other putative foodborne pathogens classically screened for during FBO investigations: *Salmonella* spp, *Staphylococcus aureus*, *Clostridium perfringens* and enteric viruses when symptoms occurred more than 24 h after ingestion of suspected food. The collected data revealed that for 19/49 FBOs (38.8%), no other food pathogen could be detected (Table 1). This ranks Bt isolates first among the hypothetical etiological agents of these 19 FBOs, on the basis of current knowledge. These FBOs were attributed to group A for this study, the others being attributed to group B.

### Epidemiology of Bt-associated FBOs

According to the available epidemiologic data, more than 673 patients were affected by the 49 Bt-associated FBOs, and more than 330 patients when restricted to the 19 Bt-FBOs from group A. The symptoms reported were of the gastroenteritis type, such as diarrhea, vomiting, nausea, and abdominal pain, with a median onset time of 5 h (from 15 minutes to more than 24 h). Depending of the FBO, the rate of Bt contamination ranged from 1x10^2^ to more than 1x10^7^ CFU/g of food (median of 9x10^2^). In 44/49 FBOs (89.8%), the level of Bt contamination was found to be below the alert threshold of 1x10^5^ CFU/g established by the French Ministry of Agriculture [16].

### Bt versus non-Bt isolates in foodstuffs

**Fig 1:**
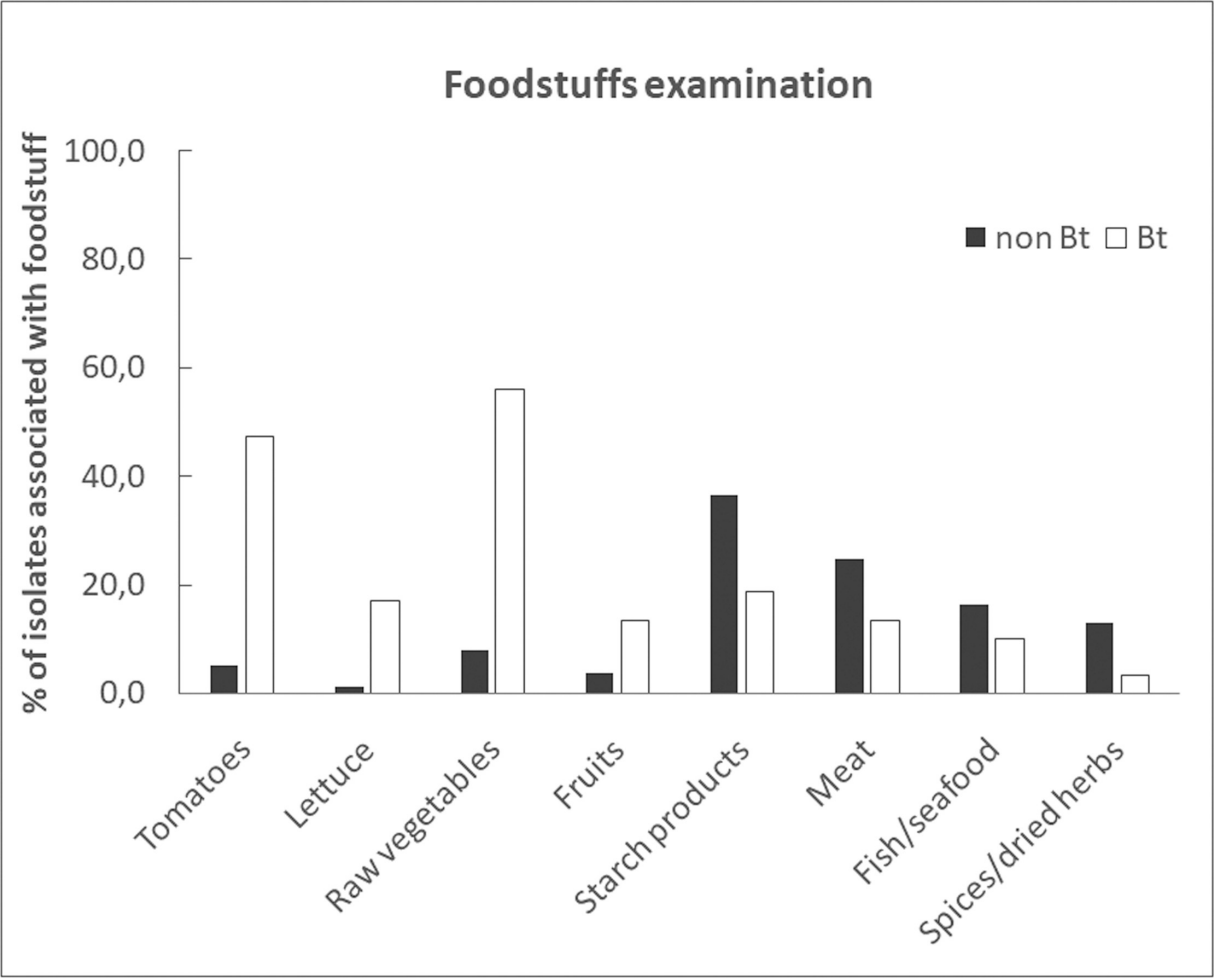
Frequency of association of Bt and non-Bt isolates with eight specific types of food. The analysis was performed from 59 representative Bt and 437 representative non-Bt isolates, collected from FBO investigations (i.e. 1 isolate per FBO, per dish/foodstuff and per genotypic and phenotypic profile). The frequencies correspond to the % of each type of isolate collected from a dish containing tomatoes (including cooked tomatoes but excluding tomato sauce), lettuce, raw vegetables, fruits (raw and cooked), starch products, meat, fish or seafood, and spices or dried herbs.

To search for food products specifically associated with Bt isolates, we compared the frequency of isolation of representative FBO-Bt and -Bc (non-Bt) isolates, from dishes containing eight types of food products (Fig 1). Our data revealed that Bt was isolated more frequently than non-Bt in the presence of raw vegetables, in particular tomatoes and lettuce (respectively 7, 9 and 15 times more), and in the presence of fruits (3 times more). By contrast, Bt was found to be less commonly associated than other Bc to starch products, meat, fish or seafood, and spices or dried herbs.

### FBO-associated Bt isolates and pesticide Bt strains

To explore the putative path of agricultural Bt contamination via pesticides, a panel of 19 phytopharmaceutical products formulated with 10 different Bt strains was collected (Table 2). After isolating their bacterial content on specific media, we compared them to FBO-associated Bt, from a selection of representative isolates (i.e. exhibiting original phenotypic and genotypic profiles per FBO and per foodstuff (Table 3, S2 Table). Almost all Bt isolates were found to be hemolytic and exhibited a lecithinase activity on Mossel plates, with the exception of one FBO-Bt (14SBCL08) and one commercial Bt (18SBCL485), found to be lecithinase-negative. All commercial Bt belonged to phylogenetic group IV, as well as 98% of FBO-Bt. We then searched, by conventional PCR, for the presence of genes encoding six virulence factors. The majority of Bt isolates (94%) exhibited a common signature, defined by the presence of *cytK2*, *nheA/B/C* and *hblC/D/A*, and the absence of *ces*, *hlyII* and *cytK1*. By contrast, the commercial strain NB-176 (isolate 18SBCL485) was *cytK2-*negative, and all commercial Bt ssp. *israelensis* and *morrisoni* strains were found to be *hlyII-*positive, as well as two FBO-Bt isolates (08CEB128BAC and 17SBCL429).

**Table 2:** List of the 10 commercial Bt strains collected for the study from 19 commercially available products, distributed into 4 different subspecies: *Aizawai*, *kurstaki*, *israelensis* and *morrisoni*.

Bacterial strain	Trade name	User	Use*	Subspecies	Corresponding ID in this study
Phytopharmaceutical products					
ABTS-1857	Xentari, Neudorff Raupenfrei, Compo Insecticida orugas	Professional / amateur	A, B	*aizawai*	18SBCL209,18SBCL210, 18SBCL449
GC-91	Agree 50 WG	Professional	A, B	*aizawai*	18SBCL617
ABTS-351	Dipel DF, Dipel DF jardin, Bactospeine DF, Foray 48B	Professional / amateur	A, B, C	*kurstaki*	18SBCL212, 18SBCL214, 18SBCL448, 18SBCL450
PB-54	Belthirul probelte jardin	Amateur	A, B	*kurstaki*	18SBCL215, 18SBCL216
SA-11	Bio-Control Pyrale du buis, Solabiol, Protecta Delfin Jardin, Delfin WG	Professional / amateur	A, B	*kurstaki*	18SBCL217, 18SBCL218, 18SBCL219, 18SBCL487
SA-12	Costar WG	Professional	A, B	*kurstaki*	18SBCL614
EG2348	Lepinox	Professional	A, B	*kurstaki*	18SBCL421
NB-176	Novodor	Professional	D	*morrisoni*	18SBCL485
Biocides					
AM65-52	Vectobac	Professional	E	*israelensis*	18SBCL484
BMP144	Aquabac	Professional	E	*israelensis*	18SBCL483

* A = various field crops, B = ornamental plants, C = forests, D = potato crops, and E = mosquito control.

**Table 3:** Phenotypic and genotypic characteristics of representative FBO- and commercial Bt isolates.

Phenotypic/genotypic characteristics	FBO-Bt (%)	Commercial Bt (%)
Lecithinase +	98	95
Hemolysis +	100	100
Phylogenetic group IV	98	100
*cytK1 +*	0	0
*cytK2 +*	97	95
*ces +*	0	0
*hlyII+*	3	16
*nhe (A/B/C)+*	100	100
*hbl (C/D/A)+*	98	100

The lecithinase and hemolysis activities were searched on specific media as described in materials and methods. The presence of virulence genes *cytK1*, *cytK2*, *ces*, *hlyII*, *nhe* (A/B/C) and *hbl* (C/D/A) was determined by conventional PCR. The assignment to phylogenetic group IV was realized by partial sequencing of the *panC* gene. The percentages were calculated from panels of 59 representative FBO-Bt isolates and 19 insecticide strains. The detailed characterization of all Bt isolates is listed in S2 Table.

### *In vitro* production of Nhe and Hbl

**Fig 2:**
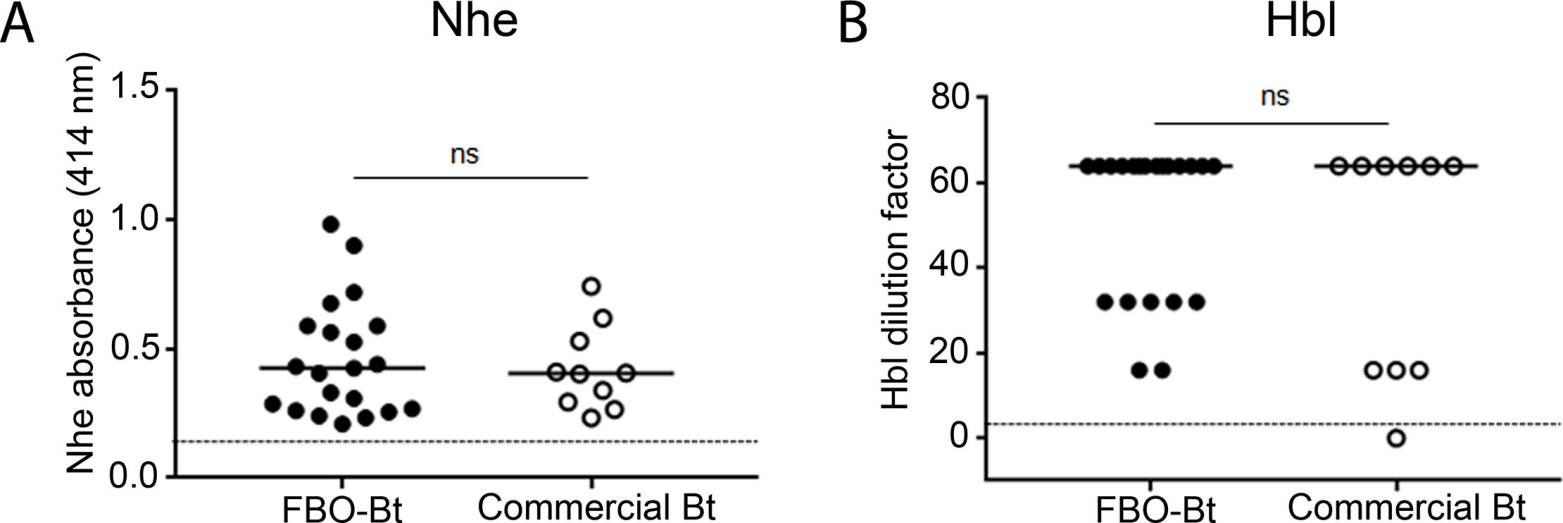
Nhe and Hbl production by a representative panel of Bt isolates composed of FBO-Bt isolates from group A (n = 21) and commercial Bt strains (n = 10). (A) Nhe production was expressed as Nhe absorbance (414nm), corresponding to the coloration intensity obtained with the BDE VIATM kit (3M-Tecra), according to the manufacturer’s recommendations. (B) Hbl dilution values correspond to the highest dilution for which Hbl remained detectable, using the immunoenzymatic kit BCET-RPLA (Oxoid), and according to the manufacturer’s recommendations. Dilution = 1 is the limit of detection of Hbl and corresponds to 2 ng/mL of Hbl components. The value indices obtained for FBO-Bt and commercial Bt were compared using the non-parametric Wilcoxon statistical test. ns = not significant.

After detecting the presence of *nhe* and *hbl* for most of Bt isolates, we assessed their expression *in vitro* by detection of toxin components in culture supernatants. The experiment was performed with a panel of 31 isolates (composed of 21 Bt isolated from 19 FBOs from group A and 10 commercial Bt), using two immunological tests (Fig 2, S3 Table). Results indicated that the Nhe production indices of Bt isolates from FBO and pesticides were analogous (i.e. not significantly different, p-value = 0.9003), with respective Nhe absorbance medians of 0.425 and 0.405. Likewise, the Hbl production levels of FBO-Bt isolates was found to be comparable to the one of pesticide Bt (median Hbl dilution = 1/64 in both cases, p-value = 0.4168). The 18SBCL483A isolate (commercial Bt strain BMP144) was the only Bt isolate found to be associated with a level of Hbl production below the detection limit in tested conditions.

### M13-PCR typing

**Fig 3:**
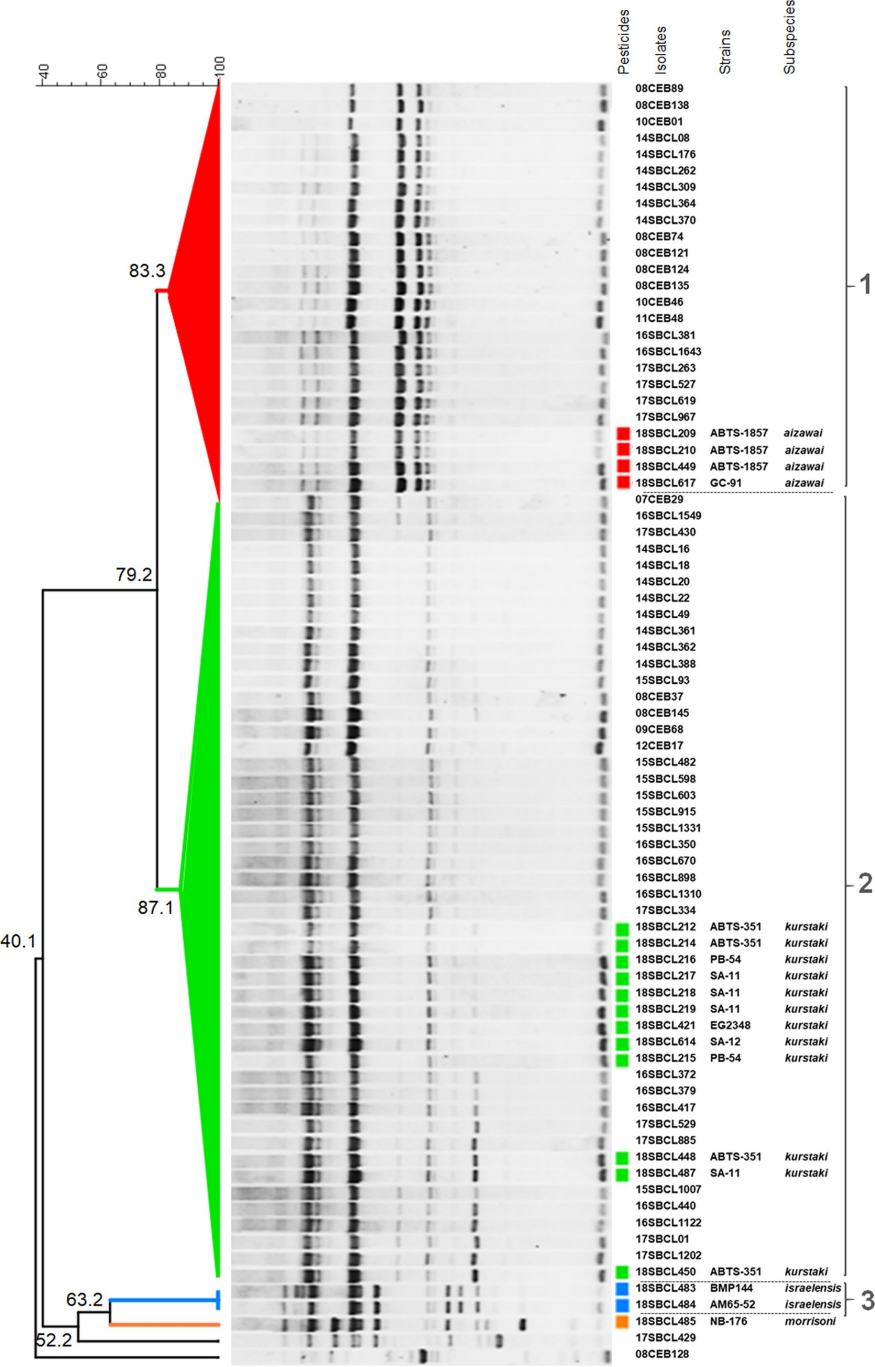
Dendrogram of representative FBO- and commercial Bt isolates based on M13-PCR typing data. After migration of M13-PCR products onto 1% agarose gels, the acquired images were analyzed with Bionumerics 7.6. The dendrogram was constructed using Dice coefficients and UPGMA clustering, with tolerance and optimisation set at 1%. Three groups of isolates were defined (1 to 3) based on a calculated similarity level above 80%.

With the aim of comparing Bt isolates more precisely, they were analyzed by M13-PCR typing and Dice’s coefficient similarity calculations (Fig 3). Based on a similarity threshold of 80%, commonly used for this type of method with Bc [32, 45], Bt isolates were clustered into three groups named 1 to 3. Regarding the commercial strains, Bt isolates were grouped according to their subspecies, as follows. Group 1 was associated with commercial Bt ssp. *aizawai* (strains ABTS-1857 and GC-91), Group 2 with commercial Bt ssp.*kurstaki* (strains ABTS-351, PB-54, SA-11, SA-12 and EG2348), and Group 3 with commercial Bt ssp.*israelensis* (strains AM65-52 and BMP144). Furthermore, the discrimination seemed restricted to the subspecies level, since distinct commercial strains belonging to the same subspecies could not be distinguished within the groups 1 to 3 by this typing method. Among the 59 representative FBO-Bt isolates, 21 (35.6%) were found to be associated with the Group 1 and 36 (61%) with Group 2, suggesting they could be attributed to the corresponding subspecies *aizawai* and *kurstaki*, respectively. Taken together, 96.6% of FBO-Bt isolates were found to be indistinguishable from commercial Bt ssp. *kurstaki* or *aizawai* by M13-PCR. No FBO isolates were found to be associated with Group 3 (attributed to ssp. *israelensis*), and two isolates (08CEB128BAC and 17SBCL429) were found to be unclustered, as well as the commercial Bt strain NB-176 (ssp. *morrisoni*).

### Genomic relationships between Bt isolates from FBO and commercial products

**Fig 4:**
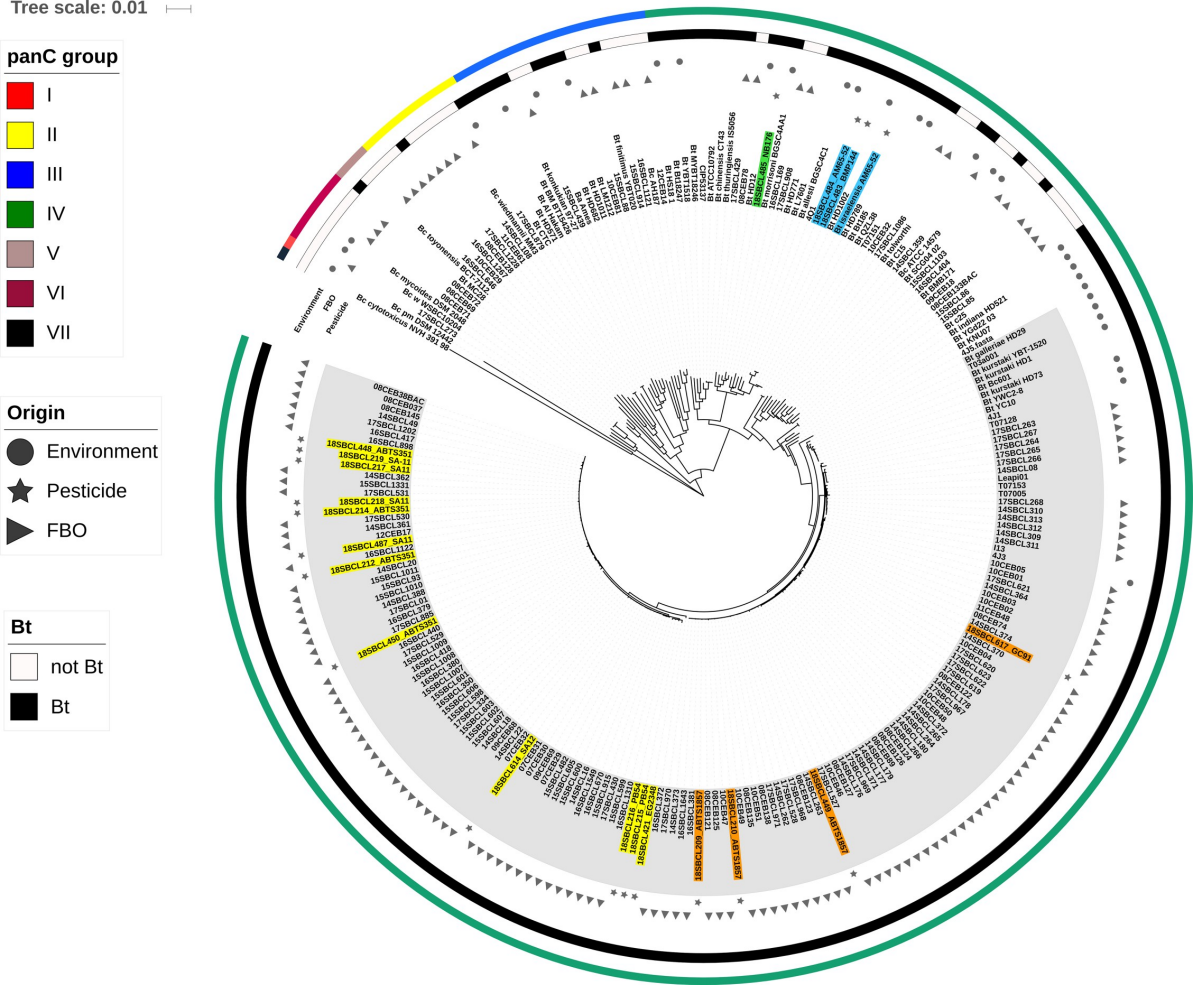
K-mer based phylogeny of 234 Bc/Bt isolates. The dataset was composed of 208 *Bacillus thuringiensis* isolates. Among them, 153 were collected from FBOs, 19 from pesticides, and 32 from the environment (soils or insects). Genomic sequences were obtained in this study after genomic DNA extraction and NGS for 172 isolates, or collected from the NCBI database for 62 isolates. Detailed information related to each isolate is listed in S4 Table. The phylogenic tree was obtained with an in-house script using Mash as distance estimation and neighbor-joining (NJ) as the clustering method. Tree visualization was performed with iTOL v5 [46]. The IDs of Bt strains isolated from biopesticides were highlighted according to their respective subspecies (green: *morrisoni*, blue: *israelensis*, yellow: *kurstaki*, and orange: *aizawai*). The genome IDs from the dominant clade was highlighted in grey.

To further compare Bt isolates, we carried out analyses of their whole genomes. The DNA of 172 isolates (i.e. 152 FBO-associated strains, 19 insecticide strains and one reference strain) was purified and sequenced before genome assembly. To evaluate their genomic relatedness within the Bc group, a phylogenetic tree based on shared k-mers was built, together with 62 publicly available sequences of various Bc strains (Fig 4). Most genomes were found to be organized according to their phylogenetic group based on *panC* sequencing; groups I and IV were the most distant., Interestingly, the k-mer phylogeny revealed a dominant clade (highlighted in grey) composed of 144 closely related genomes of group IV Bt isolates. Except for two strains (08CEB128 and 17SBCL429), this clade included all the FBO-associated Bt analyzed in the present study. They included all the collected commercial strains from ssp. *aizawai* and *kurstaki*. By contrast, commercial Bt isolates from ssp. *israelensis* and *morrisoni* were located out of this clade.

### Main clusters of FBO and commercial Bt isolates

**Fig 5:**
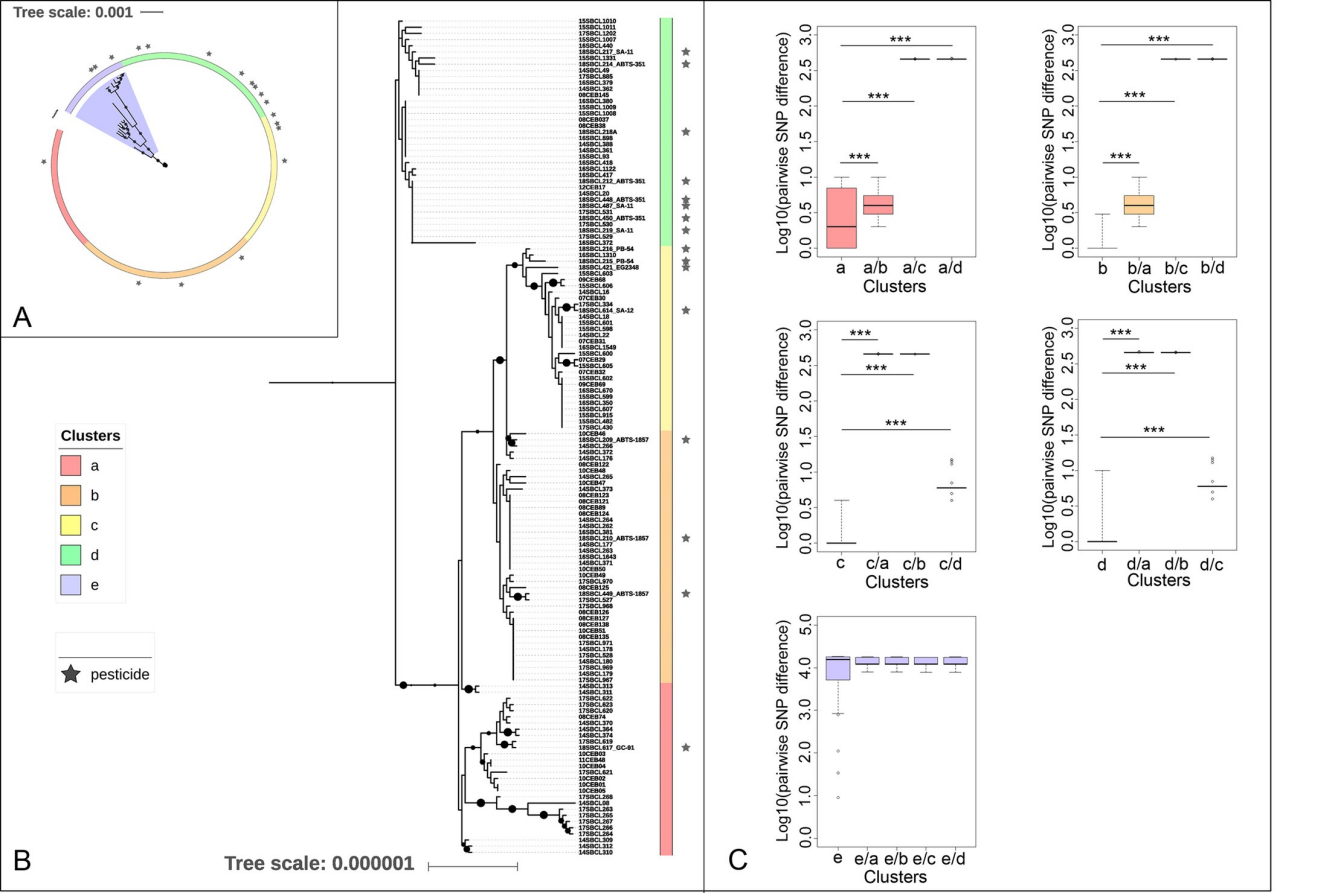
Core-genome phylogenic analysis of 154 Bc/Bt isolated from FBOs and commercial products. The analysis was performed using the pipeline iVARCall2 [41], and HD73 as a reference Bt strain. For better visualization, the tree (B) was built from the main one (A), excluding the cluster e, composed of 17 distant isolates. Phylogenetic reconstructions were performed by maximum likelihood with K3P model of substitution based on IQtree [43] and visualization of trees was performed with iTOL v5 [46]. (B) The trees were re-rooted on a branch of cluster e (A) and on a branch of cluster d (B). Bootstraps from 80 to 100 were represented by proportionately sized circles. Based on their cluster distribution (a to e), isolates were associated with red, orange, yellow, green and blue color, respectively. (C) Distribution of intra- (i.e. a, b, c, d or e) and inter-cluster cluster (i.e. a/b, a/c, a/d, b/a, b/c, b/d, c/a, c/b, c/d, d/a, d/b, d/c, e/a, e/b, e/c, e/d) pairwise SNP distances. Distances were compared with Wilcoxon rank-sum test implemented in R. *** = *p*-value<0.001.

**Fig 6:**
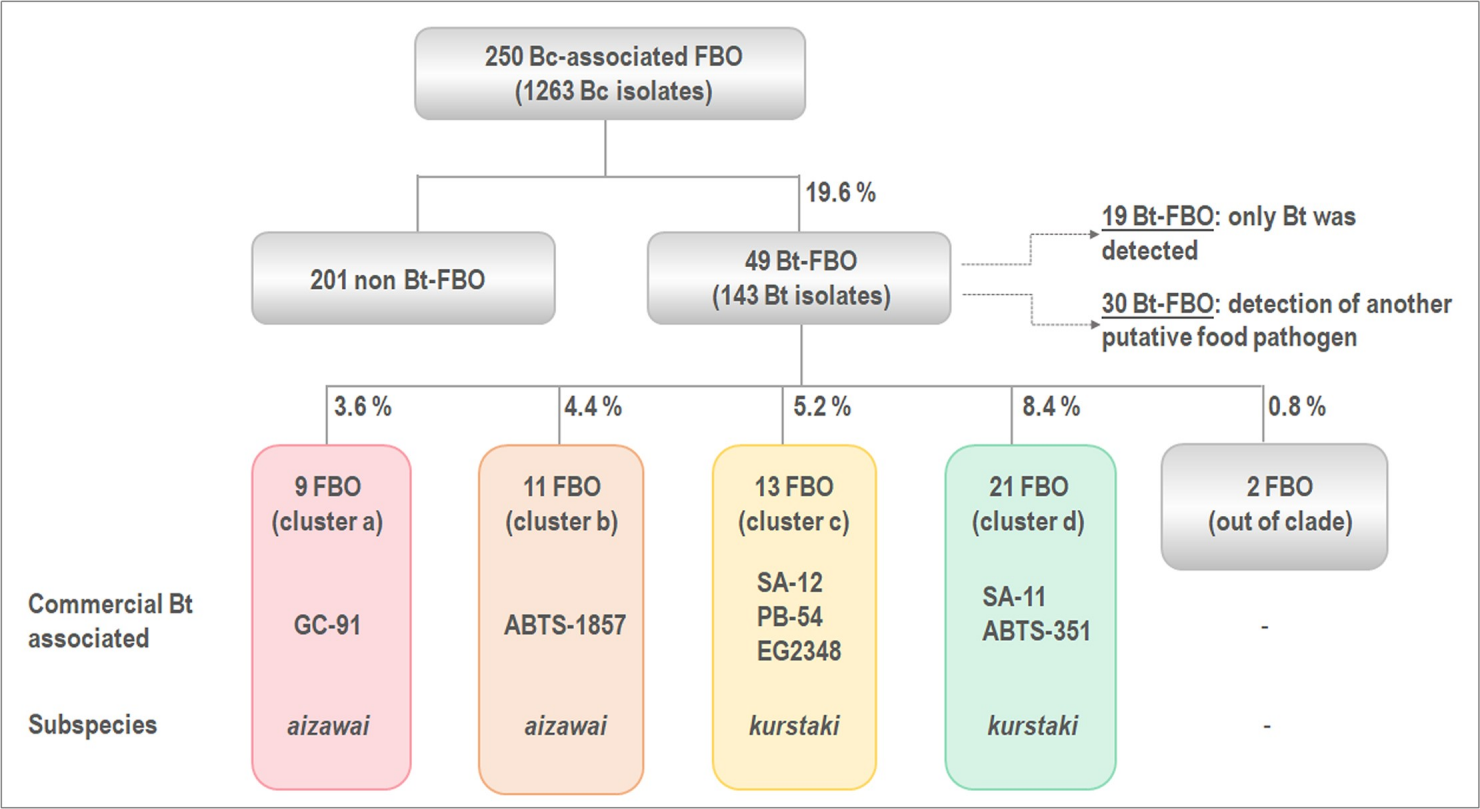
Diagram depicting the distribution of Bc-associated samples characterized in this study. From 250 toxic episodes, at least one Bt was isolated in the context of 49 toxic events (i.e. 20%). Based on the genetic relatedness of corresponding isolates, FBOs were classified into 4 clusters (a to d). Nine FBOs were associated the insecticide strain GC-91 within the cluster a, 11 with the insecticide strain ABTS-1857 within the cluster b, 13 with Bt *kurstaki* SA-12, PB-54 and EG-2348 within the cluster c, and 21 with SA-11 and ABTS-351 within the cluster d.

To refine and consolidate the k-mer-based phylogeny, we performed an SNP-based analysis of the core genome of 154 isolates from group IV, comprising all commercial Bt isolates and all FBO-associated Bt isolates (except for 08CEB128 because of its large genetic distance). From a new phylogenetic reconstruction (Fig 5A) and the computed pairwise SNP distances (not shown), we highlighted the presence of 5 clusters (named a to e). Cluster e, that grouped together 17 isolates distant from the rest of the dataset, was removed from the tree for better visualization (Fig 5B). In agreement with the k-mer phylogeny, commercial Bt isolates *aizawai* and *kurstaki* were scattered among Bt isolated from 47 toxic episodes, and distributed into clusters a to d. Clusters a and b comprised isolates related to 9 and 11 FBOs, respectively along with commercial Bt strains GC-91 (cluster a), and ABTS-1857 (cluster b). Cluster c included 13 FBOs with pesticides SA-12, PB-54 and EG2348. Lastly, the commercial strains SA-11 and ABTS-351 were located in cluster d, as well as Bt associated with 21 FBOs (Fig 6). In the case of 4 outbreaks (23, 33, 36 and 45), Bt isolates were dispatched into 2 distinct clusters, suggesting the presence of two Bt populations. Moreover, this distribution was confirmed with a phylogenetic reconstruction built from clustering of the accessory genomes (S2 Fig). The pairwise SNP distances between Bt isolates ranged from 0 to 10 SNP within a same cluster, with median distances of 2, 0, 1 and 1 SNP for clusters a to d, respectively. The inter-group distances were found to be significantly higher than intra-group SNP distances for each cluster (Fig 5C). Collectively, our data highlighted a common genetic background between commercial strains from ssp *aizawai* and *kurstaki* and isolates related to 47 FBOs, corresponding to 18.8% of Bc-associated FBOs (Fig 6). However, insecticides from subspecies *israelensis* and *morrisoni* were located out of the four defined clusters a to d, along with two FBO-Bt isolates.

## Discussion

Because of a lack of investigations, the involvement of Bt in FBOs is most likely underestimated. To explore this hypothesis, we screened for the presence of Bt within the largest collection of FBO-associated Bc isolates ever analyzed to our knowledge. We report the detection of at least one type of Bt isolate in the context of 49 FBOs, out of 250 Bc-associated FBOs analyzed (19.6%). To address the question of whether Bt could be responsible for the toxic events, the presence of other food pathogens was verified from collected data. In about 39% of Bt-associated FBOs, no other pathogenic microorganism could be detected, except for Bt, making it the most likely causal agent. However, we cannot completely exclude the presence of another microorganism that may not have been found through *in vitro* detection, but this assumption would concern a relatively high number of toxic episodes (19 FBOs from that point), which seems unlikely.

The 49 Bt-associated FBOs affected more than 673 people in total in France between 2007 and 2017, and caused symptoms such as diarrhea and vomiting. Vomiting is commonly considered the result of the Bc emetic toxin, cereulide [47]. Nevertheless, none of the identified Bt isolates were positive for the detection of *ces*, suggesting the reported vomiting-type symptoms could be correlated with another virulence marker, potentially not described so far. Additionally, almost all Bt isolates harbored genes encoding three major enterotoxins (HblC/D/A, NheA/B/C, and CytK2), predicted to generate diarrhea-type symptoms. Interestingly, Guinebretiere and co-authors showed that the co-occurrence of *cytK*, *nhe* and *hbl* was significantly more often associated with food-poisoning Bc strains than food-related Bc strains [34]. According to previous studies [34], the levels of Nhe and Hbl production of Bt isolates measured in this study are intermediate, when compared with the whole Bc group. However, in another study carried out by the same authors, Bc isolates from phylogenetic group IV-2 (*nhe+*, *cytk+* and *hbl+)* were found to be associated with elevated cytotoxicity levels on Caco2 cells [48]. This suggests that these enterotoxins are not fully responsible for the cytotoxic damage linked to Bc isolates, and calls again to screen for other virulence markers, previously described or not, along with the cytotoxicity assessment of Bt isolates characterized in this study.

Bt were enumerated from foodstuffs with a median value close to 1x10^3^ CFU/g of food, and for about 90% of FBOs, the enumerations were found to be below the alert threshold established by the French Ministry (10^5^ CFU/g), concerning bacteria from the whole Bc group. The infective dose of Bc is considered to mainly range from 10^5^ to 10^8^ CFU/g [49]. Nevertheless, this dose is regularly questioned since it can vary considerably from an outbreak to another one. For instance, low doses of Bc (2.10^2^ to 1.10^3^ CFU/g) have also been described in foods causing diseases [21, 29]. One possible explanation would be that the aggregation of Bc spores strongly interfere with the enumeration of bacilli [21]. Moreover, the infective dose of spores in diarrheal diseases is predicted to be lower than the one of vegetative cells, due to their resistance to the acidic gastric environment [50]. Therefore, our results are consistent with previous findings and suggest that the alert threshold might need to be re-evaluated.

In more than half of cases, the foodstuffs from which Bt were isolated were composed of raw food and in particular tomatoes, whereas non-Bt isolates were 7 to 9 times less frequently associated with raw food. These results are consistent with the Bt prevalence estimations made by Kim et al. who detected Bt in 77% of tested organic vegetables [51]. Similarly, previous studies have demonstrated the presence of high counts (above 10^4^ CFU/g) of Bt on fresh vegetables originating from Denmark [52], and some of them were undistinguishable by low resolution genotyping methods from commercial strains such as ABTS-351 [25], suggesting they could be residues of Bt insecticides. Tomatoes and lettuce do not need to be peeled before consumption, contrary to other raw vegetables, such as cucumber. This might explain why these type of food were more frequently incriminated in Bt-FBO, compared to vegetables that are subjected to the same Bt treatments. Taken together, these data reinforce the hypothesis that Bt food contamination may be of agricultural origin. However, we found that in some cases, Bt was reported to be isolated from foodstuffs not subjected to Bt treatments, e.g. Bt isolate 08CEB074, isolated from smoked salmon in FBO 3. The lack of information related to the exact composition of mixed dishes, as well as putative cross contamination between foodstuffs during food processing could easily explain this observation.

In fact, Bt ssp. *kurstaki* and *aizawai* are the most widely used bacterial insecticides [53], in particular for the treatment of various crops (including solanaceaous vegetables and cucurbits) and tree fruits [6]. To explore this putative path of contamination, we compared Bt isolated from FBOs to a representative panel of Bt pesticides. All FBO-Bt except for two, appeared to share common features with commercial Bt *aizawai* and *kurstaki* strains, including the presence of genes encoding the enterotoxins *cytK2*, *nheA/B/C* and *hblC/D/A*. By contrast, insecticidal Bt ssp. *israelensis* (BMP144 and AM65-52) and *morrisoni* (NB-176) are different due to specific genetic signatures, suggesting that they are distinct from FBO-Bt. These results are consistent with the M13-PCR characterization, highlighting similarities of pattern between pesticides belonging to ssp. *kurstaki* and *aizawai*, and about 97% of Bt isolates coming from FBOs. Nonetheless, this RAPD-type PCR is a rapid approach for routine comparison of Bc isolates and did not allow us to differentiate commercial Bt strains beyond the subspecies level, leading us to use a genomic approach for further discrimination.

A preliminary phylogeny based on shared k-mers suggested that most FBO-Bt strains we studied were embedded in a major clade of genetically close isolates (phylogenetic group IV), along with a few publicly available genomes of Bt isolated from insects. The isolates from this clade were genetically more distant from isolates from phylogenetic groups VII, then I, VI, V, II and III, successively, which is consistent with previous findings published by A. Bazinet [8]. However, the inclusion of public sequences in the dataset illustrates that the Bt species are diverse and are distributed among four different *panC* groups (i.e. II, III and V, in addition to IV). This suggests that the panel of FBO-Bt strains analyzed in this study does not reflect the full diversity of environmental Bt isolates, but appears rather to be associated with genetically close subspecies.

Based on an SNP variant analysis, we highlighted that Bt associated with 47 FBOs were specifically organized into four clusters, along with seven pesticide Bt strains belonging to ssp. *kurstaki* and *aizawai*. Since the variant calling implemented is restricted to the core genome, we confirmed this distribution with a complementary tree built from accessory genomes. Whereas new tools in WGS analysis have greatly facilitated the investigation on sources of foodborne illnesses, the elaboration of precise guidelines for interpretations remains a complex issue [54, 55]. In particular, applying core genome SNP thresholds appears insufficient since the bacterial genome plasticity can result in mutations depending of culture and isolation conditions [56]. Even if authors estimated that pairwise SNP distances between related genomes range from 0 to 4 for *Salmonella enterica* [54] and Shiga toxin-producing *E*. *coli* [57], this statement is not true for long term outbreaks where the outbreak duration and evolution rate induce increasing of pairwise SNP distances between related genomes [55]. Nevertheless, the low computed pairwise SNP distances for Bt isolates within clusters defined in the present study (0 to 2 median pairwise SNP distances) suggested that the isolates were genetically similar and likely originated from a same source. It would be useful for further analysis to investigate the evolutionary capacity of commercial Bt strains, with complementary genomic and laboratory approaches, in order to elaborate interpretation guidelines.

Three pesticide genomes were found to be distinct from FBO-Bt. The corresponding ssp. *israelensis* and *morrisoni* are respectively dedicated to control of mosquito larvae and to the protection of potato crops by spraying of the foliage, a part of the plant that is not consumed. Again, the fact that these insecticide strains are distinguishable from FBO-Bt strains is consistent with the hypothesis of an agricultural origin for Bt- associated FBOs. Bc is a ubiquitous and telluric organism; its presence in soils as a natural contaminant is also a possible hypothesis. Our results indicate that Bt was often isolated in the presence of tomatoes, for instance, which are usually cultivated relatively far from the soil. In the hypothetical case of contamination from soil due to raindrop impacts, it seems unlikely that the natural presence of Bt in soils would allow detection of high amounts of bacteria on tomatoes (about 10^3^ CFU/g).

## Conclusions

This study reported the involvement of Bt in 8 to 20% of Bc-associated FBOs considered for the study, with Bc as the second most common cause of bacterial FBOs in France. Moreover, multiple analyses converged towards the putative agricultural origin of the majority of the identified Bt isolates. Hence, given the high degree of similarity observed between FBO-associated and some commercial Bt isolates, and in the absence of contradictory evidence, we cannot rule out that pesticide Bt may have pathogenic potential. This emphasizes the need for a better understanding of the pathogenicity potential of this agent for non-target organisms, along with the development of specific monitoring tools for Bt traceability in food.

## Supporting information

S1 Table Detailed methods used for the genotypic characterization of Bc isolates. (A) List of the primers used for the study. (B) Conditions of respective PCR amplifications.
(PDF)

S2 Table List of phenotypic and genotypic characteristics of 59 representative FBO-Bt isolates and 19 pesticide Bt strains. +/- = detection/no detection of corresponding activity or gene. The phylogenetic groups were assigned according to the partial sequencing of *panC* [9]. The attribution to M13 groups (named 1 to 3) was established in this study based on similarity Dice coefficients calculated with Bionumerics.
(PDF)

S3 Table Nhe absorbance and Hbl dilution index values of a panel of FBO- and commercial Bt. * The Nhe absorbance corresponds to the coloration intensity, obtained with the BDE VIATM kit (3M-Tecra), according to the manufacturer’s recommendations. In the tested conditions, the limit of detection corresponds to Nhe Absorbance (414nm) = 0.2. ** The Hbl dilution values correspond to the highest dilution for which Hbl remained detectable, using the immunoenzymatic kit BCET-RPLA (Oxoïd), and according to the manufacturer’s recommendations. nd = not detected. Dilution = 1 is the limit of detection of Hbl and corresponds to a detection of 2ng/ml of Hbl components.
(PDF)

S4 Table List of 234 isolates used for the k-mer phylogeny. ^a^ the attribution to *Bacillus thuringiensis* (Bt) species was determined by detection of parasporal crystals by phase-contrast microscopy. ^b^ the phylogenetic clustering was based on *panC* sequences similarities [9]. ^c^ All the sequencing data obtained from this study are associated with the BioProject PRJNA547495. ^d,e^ The strains used for genome assemblies and the SNP calling analysis are indicated with a “x” in the columns “reference assemblies” and “iVARCall2”, respectively.
(PDF)

S1 Fig Production of parasporal crystals by Bt strains isolated from FBOs after 24, 48 and 72 hours of culture on sporulating HCT 0.3% Glc media. Images were acquired by phase-contrast microscopy. Strains ATCC14579 and CIP53137 were used as negative and positive controls, respectively. Red arrows indicate the presence of crystals.
(TIF)

S2 Fig Accessory phylogeny of 154 Bc/Bt FBO-associated and pesticide isolates. (A) Accessory binary genes tree, established with ROARY [44] and iQtree [43]. The visualization was done using iTOL [46]. (B) Diagrams of the distributions of intra- and inter-clusters log_10_ (number of different genes).
(PDF)

S1 Raw images (TIF)

## References

[1] Hannay CL, Fitz-James P. The protein crystals of Bacillus thuringiensis Berliner. Can J Microbiol. 1955;1(8):694–710. Epub 1955/10/01. 10.1139/m55-083 . 13270146

[2] Schnepf E, Crickmore N, Van Rie J, Lereclus D, Baum J, Feitelson J, et al Bacillus thuringiensis and its pesticidal crystal proteins. Microbiol Mol Biol Rev. 1998;62(3):775–806. Epub 1998/09/08. 9729609 10.1128/mmbr.62.3.775-806.1998 PMC98934

[3] Hofte H, Whiteley HR. Insecticidal crystal proteins of Bacillus thuringiensis. Microbiol Rev. 1989;53(2):242–55. Epub 1989/06/01. 2666844 10.1128/mr.53.2.242-255.1989 PMC372730

[4] Crickmore N, Zeigler DR, Feitelson J, Schnepf E, Van Rie J, Lereclus D, et al Revision of the nomenclature for the Bacillus thuringiensis pesticidal crystal proteins. Microbiol Mol Biol Rev. 1998;62(3):807–13. Epub 1998/09/08. 9729610 10.1128/mmbr.62.3.807-813.1998 PMC98935

[5] Zhang Q, Hua G, Adang MJ. Effects and mechanisms of Bacillus thuringiensis crystal toxins for mosquito larvae. Insect Sci. 2017;24(5):714–29. Epub 2016/09/16. 10.1111/1744-7917.12401 . 27628909

[6] Lacey LA, Grzywacz D, Shapiro-Ilan DI, Frutos R, Brownbridge M, Goettel MS. Insect pathogens as biological control agents: Back to the future. J Invertebr Pathol. 2015;132:1–41. Epub 2015/08/01. 10.1016/j.jip.2015.07.009 . 26225455

[7] Carroll LM, Cheng RA, Kovac J. No Assembly Required: Using BTyper3 to Assess the Congruency of a Proposed Taxonomic Framework for the Bacillus cereus Group With Historical Typing Methods. Front Microbiol. 2020;11:580691 Epub 2020/10/20. 10.3389/fmicb.2020.580691 33072050 PMC7536271

[8] Bazinet AL. Pan-genome and phylogeny of Bacillus cereus sensu lato. BMC Evol Biol. 2017;17(1):176 10.1186/s12862-017-1020-1 28768476 PMC5541404

[9] Guinebretiere MH, Thompson FL, Sorokin A, Normand P, Dawyndt P, Ehling-Schulz M, et al Ecological diversification in the Bacillus cereus Group. Environ Microbiol. 2008;10(4):851–65. Epub 2007/11/27. 10.1111/j.1462-2920.2007.01495.x . 18036180

[10] SPF. Santé Publique France. "Surveillance des toxi-infections alimentaires collectives—Données de la déclaration obligatoire, 2017". 2019.

[11] EFSA. The European Union summary report on trends and sources of zoonoses, zoonotic agents and food-borne outbreaks in 2017. 2018. 10.2903/j.efsa.2018.5500 PMC7009540 32625785

[12] Callegan MC, Kane ST, Cochran DC, Novosad B, Gilmore MS, Gominet M, et al Bacillus endophthalmitis: roles of bacterial toxins and motility during infection. Invest Ophthalmol Vis Sci. 2005;46(9):3233–8. Epub 2005/08/27. 10.1167/iovs.05-0410 . 16123424

[13] Glasset B, Herbin S, Granier SA, Cavalie L, Lafeuille E, Guerin C, et al Bacillus cereus, a serious cause of nosocomial infections: Epidemiologic and genetic survey. PLoS One. 2018;13(5):e0194346 Epub 2018/05/24. 10.1371/journal.pone.0194346 29791442 PMC5966241

[14] Lotte R, Herisse AL, Berrouane Y, Lotte L, Casagrande F, Landraud L, et al Virulence Analysis of Bacillus cereus Isolated after Death of Preterm Neonates, Nice, France, 2013. Emerg Infect Dis. 2017;23(5):845–8. Epub 2017/04/19. 10.3201/eid2305.161788 28418291 PMC5403044

[15] EC. European Commission. Commission regulation N° 2073/2005 of 15 November 2005 on microbiological criteria for foodstuffs. 2005.

[16] DGAL. French Ministry of Agriculture. « Révision et publication du Guide de gestion des alertes d’origine alimentaire entre les exploitants de la chaîne alimentaire et l’administration lorsqu’un produit ou un lot de produits est identifié ». 2009;DGAL/MUS/N2009-8188, 2009.

[17] Ramarao N, Sanchis V. The pore-forming haemolysins of bacillus cereus: a review. Toxins (Basel). 2013;5(6):1119–39. 10.3390/toxins5061119 23748204 PMC3717773

[18] Lund T, De Buyser ML, Granum PE. A new cytotoxin from Bacillus cereus that may cause necrotic enteritis. Mol Microbiol. 2000;38(2):254–61. 10.1046/j.1365-2958.2000.02147.x . 11069652

[19] Hardy SP, Lund T, Granum PE. CytK toxin of Bacillus cereus forms pores in planar lipid bilayers and is cytotoxic to intestinal epithelia. FEMS Microbiol Lett. 2001;197(1):47–51. 10.1111/j.1574-6968.2001.tb10581.x . 11287145

[20] Fagerlund A, Ween O, Lund T, Hardy SP, Granum PE. Genetic and functional analysis of the cytK family of genes in Bacillus cereus. Microbiology. 2004;150(Pt 8):2689–97. Epub 2004/08/04. 10.1099/mic.0.26975-0 . 15289565

[21] Stenfors Arnesen LP, Fagerlund A, Granum PE. From soil to gut: Bacillus cereus and its food poisoning toxins. FEMS Microbiol Rev. 2008;32(4):579–606. Epub 2008/04/22. 10.1111/j.1574-6976.2008.00112.x . 18422617

[22] Mathur A, Feng S, Hayward JA, Ngo C, Fox D, Atmosukarto II, et al A multicomponent toxin from Bacillus cereus incites inflammation and shapes host outcome via the NLRP3 inflammasome. Nat Microbiol. 2019;4(2):362–74. 10.1038/s41564-018-0318-0 . 30531979 PMC7685251

[23] Agata N, Ohta M, Mori M, Isobe M. A novel dodecadepsipeptide, cereulide, is an emetic toxin of Bacillus cereus. FEMS Microbiol Lett. 1995;129(1):17–20. 10.1016/0378-1097(95)00119-P . 7781985

[24] Gaviria Rivera AM, Granum PE, Priest FG. Common occurrence of enterotoxin genes and enterotoxicity in Bacillus thuringiensis. FEMS Microbiol Lett. 2000;190(1):151–5. Epub 2000/09/12. 10.1111/j.1574-6968.2000.tb09278.x . 10981706

[25] Rosenquist H, Smidt L, Andersen SR, Jensen GB, Wilcks A. Occurrence and significance of Bacillus cereus and Bacillus thuringiensis in ready-to-eat food. FEMS Microbiol Lett. 2005;250(1):129–36. Epub 2005/07/27. 10.1016/j.femsle.2005.06.054 . 16043311

[26] McIntyre L, Bernard K, Beniac D, Isaac-Renton JL, Naseby DC. Identification of Bacillus cereus group species associated with food poisoning outbreaks in British Columbia, Canada. Appl Environ Microbiol. 2008;74(23):7451–3. Epub 2008/10/14. 10.1128/AEM.01284-08 18849447 PMC2592946

[27] EFSA. BIOHAZ Panel. Risks for public health related to the presence of Bacillus cereus and other Bacillus spp. including Bacillus thuringiensis in foodstuffs. 2016.

[28] Johler S, Kalbhenn EM, Heini N, Brodmann P, Gautsch S, Bagcioglu M, et al Enterotoxin Production of Bacillus thuringiensis Isolates From Biopesticides, Foods, and Outbreaks. Front Microbiol. 2018;9:1915 Epub 2018/09/08. 10.3389/fmicb.2018.01915 30190709 PMC6115515

[29] Glasset B, Herbin S, Guillier L, Cadel-Six S, Vignaud ML, Grout J, et al Bacillus cereus-induced food-borne outbreaks in France, 2007 to 2014: epidemiology and genetic characterisation. Euro Surveill. 2016;21(48). Epub 2016/12/10. 10.2807/1560-7917.ES.2016.21.48.30413 27934583 PMC5388111

[30] Lecadet MM, Blondel MO, Ribier J. Generalized transduction in Bacillus thuringiensis var. berliner 1715 using bacteriophage CP-54Ber. J Gen Microbiol. 1980;121(1):203–12. Epub 1980/11/01. 10.1099/00221287-121-1-203 . 7252480

[31] Altschul SF, Gish W, Miller W, Myers EW, Lipman DJ. Basic local alignment search tool. J Mol Biol. 1990;215(3):403–10. Epub 1990/10/05. 10.1016/S0022-2836(05)80360-2 . 2231712

[32] Guinebretiere MH, Nguyen-The C. Sources of Bacillus cereus contamination in a pasteurized zucchini puree processing line, differentiated by two PCR-based methods. FEMS Microbiol Ecol. 2003;43(2):207–15. Epub 2003/03/01. 10.1111/j.1574-6941.2003.tb01060.x . 19719681

[33] Dice LR. Measures of the Amount of Ecologic Association Between Species. Ecology. 1945;26(3):297–302.

[34] Guinebretiere MH, Broussolle V, Nguyen-The C. Enterotoxigenic profiles of food-poisoning and food-borne Bacillus cereus strains. J Clin Microbiol. 2002;40(8):3053–6. Epub 2002/08/01. 10.1128/jcm.40.8.3053-3056.2002 12149378 PMC120679

[35] Mahamat Abdelrahim A, Radomski N, Delannoy S, Djellal S, Le Negrate M, Hadjab K, et al Large-Scale Genomic Analyses and Toxinotyping of Clostridium perfringens Implicated in Foodborne Outbreaks in France. Front Microbiol. 2019;10:777 Epub 2019/05/07. 10.3389/fmicb.2019.00777 31057505 PMC6481350

[36] Ondov BD, Treangen TJ, Melsted P, Mallonee AB, Bergman NH, Koren S, et al Mash: fast genome and metagenome distance estimation using MinHash. Genome Biol. 2016;17(1):132 Epub 2016/06/22. 10.1186/s13059-016-0997-x 27323842 PMC4915045

[37] Bankevich A, Nurk S, Antipov D, Gurevich AA, Dvorkin M, Kulikov AS, et al SPAdes: a new genome assembly algorithm and its applications to single-cell sequencing. J Comput Biol. 2012;19(5):455–77. Epub 2012/04/18. 10.1089/cmb.2012.0021 22506599 PMC3342519

[38] Bosi E, Donati B, Galardini M, Brunetti S, Sagot MF, Lio P, et al MeDuSa: a multi-draft based scaffolder. Bioinformatics. 2015;31(15):2443–51. Epub 2015/03/27. 10.1093/bioinformatics/btv171 . 25810435

[39] Kosugi S, Hirakawa H, Tabata S. GMcloser: closing gaps in assemblies accurately with a likelihood-based selection of contig or long-read alignments. Bioinformatics. 2015;31(23):3733–41. Epub 2015/08/12. 10.1093/bioinformatics/btv465 . 26261222

[40] Letunic I, Bork P. Interactive Tree Of Life (iTOL) v4: recent updates and new developments. Nucleic Acids Res. 2019;47(W1):W256–W9. Epub 2019/04/02. 10.1093/nar/gkz239 30931475 PMC6602468

[41] Felten A, Vila Nova M, Durimel K, Guillier L, Mistou MY, Radomski N. First gene-ontology enrichment analysis based on bacterial coregenome variants: insights into adaptations of Salmonella serovars to mammalian- and avian-hosts. BMC Microbiol. 2017;17(1):222 10.1186/s12866-017-1132-1 29183286 PMC5706153

[42] Didelot X, Wilson DJ. ClonalFrameML: efficient inference of recombination in whole bacterial genomes. PLoS Comput Biol. 2015;11(2):e1004041 10.1371/journal.pcbi.1004041 25675341 PMC4326465

[43] Nguyen LT, Schmidt HA, von Haeseler A, Minh BQ. IQ-TREE: a fast and effective stochastic algorithm for estimating maximum-likelihood phylogenies. Mol Biol Evol. 2015;32(1):268–74. Epub 2014/11/06. 10.1093/molbev/msu300 25371430 PMC4271533

[44] Page AJ, Cummins CA, Hunt M, Wong VK, Reuter S, Holden MT, et al Roary: rapid large-scale prokaryote pan genome analysis. Bioinformatics. 2015;31(22):3691–3. 10.1093/bioinformatics/btv421 26198102 PMC4817141

[45] Gdoura-Ben Amor M, Siala M, Zayani M, Grosset N, Smaoui S, Messadi-Akrout F, et al Isolation, Identification, Prevalence, and Genetic Diversity of Bacillus cereus Group Bacteria From Different Foodstuffs in Tunisia. Front Microbiol. 2018;9:447 Epub 2018/03/30. 10.3389/fmicb.2018.00447 29593691 PMC5858518

[46] Letunic I, Bork P. Interactive Tree Of Life (iTOL): an online tool for phylogenetic tree display and annotation. Bioinformatics. 2007;23(1):127–8. Epub 2006/10/20. 10.1093/bioinformatics/btl529 . 17050570

[47] Agata N, Mori M, Ohta M, Suwan S, Ohtani I, Isobe M. A novel dodecadepsipeptide, cereulide, isolated from Bacillus cereus causes vacuole formation in HEp-2 cells. FEMS Microbiol Lett. 1994;121(1):31–4. 10.1111/j.1574-6968.1994.tb07071.x . 8082824

[48] Guinebretiere MH, Velge P, Couvert O, Carlin F, Debuyser ML, Nguyen-The C. Ability of Bacillus cereus group strains to cause food poisoning varies according to phylogenetic affiliation (groups I to VII) rather than species affiliation. J Clin Microbiol. 2010;48(9):3388–91. Epub 2010/07/28. 10.1128/JCM.00921-10 20660215 PMC2937725

[49] Granum PE, Lund T. Bacillus cereus and its food poisoning toxins. FEMS Microbiol Lett. 1997;157(2):223–8. Epub 1998/01/22. 10.1111/j.1574-6968.1997.tb12776.x . 9435100

[50] Clavel T, Carlin F, Lairon D, Nguyen-The C, Schmitt P. Survival of Bacillus cereus spores and vegetative cells in acid media simulating human stomach. J Appl Microbiol. 2004;97(1):214–9. Epub 2004/06/10. 10.1111/j.1365-2672.2004.02292.x . 15186458

[51] Kim JB, Choi OK, Kwon SM, Cho SH, Park BJ, Jin NY, et al Prevalence and Toxin Characteristics of Bacillus thuringiensis Isolated from Organic Vegetables. J Microbiol Biotechnol. 2017;27(8):1449–56. 10.4014/jmb.1703.03063 . 28683523

[52] Frederiksen K, Rosenquist H, Jorgensen K, Wilcks A. Occurrence of natural Bacillus thuringiensis contaminants and residues of Bacillus thuringiensis-based insecticides on fresh fruits and vegetables. 2006 5 Report No.: 0099–2240 (Print) 0099–2240 (Linking) Contract No.: 5. 10.1128/AEM.72.5.3435-3440.2006 16672488 PMC1472320

[53] Casida JE, Bryant RJ. The ABCs of pesticide toxicology: amounts, biology, and chemistry. Toxicol Res (Camb). 2017;6(6):755–63. Epub 2018/08/10. 10.1039/c7tx00198c 30090540 PMC6062263

[54] Pightling AW, Pettengill JB, Luo Y, Baugher JD, Rand H, Strain E. Interpreting Whole-Genome Sequence Analyses of Foodborne Bacteria for Regulatory Applications and Outbreak Investigations. Front Microbiol. 2018;9:1482 Epub 2018/07/26. 10.3389/fmicb.2018.01482 30042741 PMC6048267

[55] Radomski N, Cadel-Six S, Cherchame E, Felten A, Barbet P, Palma F, et al A Simple and Robust Statistical Method to Define Genetic Relatedness of Samples Related to Outbreaks at the Genomic Scale—Application to Retrospective Salmonella Foodborne Outbreak Investigations. Front Microbiol. 2019;10:2413 Epub 2019/11/12. 10.3389/fmicb.2019.02413 31708892 PMC6821717

[56] Allard MW, Luo Y, Strain E, Li C, Keys CE, Son I, et al High resolution clustering of Salmonella enterica serovar Montevideo strains using a next-generation sequencing approach. BMC Genomics. 2012;13:32 Epub 2012/01/21. 10.1186/1471-2164-13-32 22260654 PMC3368722

[57] Crowe SJ, Bottichio L, Shade LN, Whitney BM, Corral N, Melius B, et al Shiga Toxin-Producing E. coli Infections Associated with Flour. N Engl J Med. 2017;377(21):2036–43. Epub 2017/11/23. 10.1056/NEJMoa1615910 29166238 PMC5792826

