# Expression of Concern: Comparative phenotypic, genotypic and genomic analyses of *Bacillus thuringiensis* associated with foodborne outbreaks in France

**DOI:** 10.1371/journal.pone.0276871

**Published:** 2023-05-01

**Authors:** The PLOS ONE Editors

After this article [1] was published, concerns were raised about the data interpretation presented in [1]. Specifically, the causal relationship reported between foodborne outbreaks (FBOs) and the presence of *Bacillus thuringiensis* (Bt) in samples taken from French FBO events between 2007 and 2017 was questioned. The concerns were evaluated by the PLOS Publication Ethics team, *PLOS ONE* senior staff members, and two members of the Editorial Board.

The following concerns with the article remain unresolved:

1. Food poisoning cases in [1], where Bt isolates with high similarity to commercial strains were isolated, were associated with vomiting symptoms typical of cereulide toxin. However, Commercial Bt strains lack the ability to produce emetic cereulide toxin.
2. The results mention “reported vomiting-type symptoms could be correlated with another virulence marker, potentially not described so far.” The Board Members commented that the only evidence linking Bt isolates to the vomiting is co-occurrence. This observation does not provide sufficient scientific base to conclude causation, especially considering the vast amount of information available on virulence factors in Bt. It seems improbable that a new virulence factor unknown to science would be behind a clear illness symptom. Furthermore, there is a long list of foodborne illness agents that cause vomiting as a symptom (for example *Campylobacter, Clostridium, Escherichia coli, Salmonella, Staphylococcus, Vibrio, Cyclospora*, noroviruses, etc) many of which were not adequately ruled out in this study. Considering these observations, the board members concluded that it would be more likely that a known, but undetected, bacterial or viral agent may be responsible for the symptoms.
3. The methods in [1] for determining the presence of other potential bacterial strains are limited. The methods do not rule out the presence of other known potential causes for FBOs like *Campylobacter*, *Listeria* or pathogenic *E*. *coli* or toxins thereof.
4. The results in [1] mention that the potential co-occurrence of other putative foodborne pathogens was screened for when symptoms occurred more than 24 h after ingestion of suspected food. Most of the symptoms described in Table 1 occurred less then 24 h after ingestion. It is unclear if in these cases a screening for other potential FBO agents mentioned above was performed.
5. The number of colonies isolated in [1] as causative Bt strains for FBO events is small and below the threshold defined by officials in France.
6. The statement in the Conclusions section “in the absence of contradictory evidence, we cannot rule out that pesticide Bt may have pathogenic potential” is not fully supported by the analysis in [1]. Both *PLOS ONE* Board members note that correlation seen between the presence of Bt and FBOs does not provide evidence for a direct role for Bt in FBOs; rather, the data suggest that when no other foodborne pathogens could be detected, one possible etiological agent might be Bt. Additionally, the widespread use of Bt in commercial agriculture applications would support an expected very high number of linked FBO events, which have not been reported to date.
7. No Bt isolates from clinical samples were used in [1], which does not support the conclusion that there is a direct correlation between clinical symptoms and Bt as the causative agent.

In response to point 3, the first author commented that the results section of the article indicates that screening of other FBO’s was performed: “To determine whether Bt isolates were the putative etiological agents of the 49 FBOs, we examined the potential co-occurrence of other putative foodborne pathogens classically screened for during FBO investigations: *Salmonella* spp, *Staphylococcus aureus, Clostridium perfringens* and enteric viruses when symptoms occurred more than 24 h after ingestion of suspected food” and further clarified that the enumeration of coagulase positive *Staphylococci* (NF EN ISO 6888), the enumeration of *Clostridium perfringens* (NF EN ISO 7937), and the detection of Staphylococcal enterotoxins (NF EN ISO 19020) have been performed in incriminated foodstuffs. In response, the Board Members commented that there are other foodborne pathogens that do not appear to have been evaluated in this study, but which could have explained the vomiting symptoms.

The authors replied to the last point in a comment posted 06 Apr 2022: “We want to emphasize that clinical samples are very rarely collected during FBO investigations, since this type of poisoning usually evolves favourably and rapidly, and does not require hospitalization for a large majority of cases. In addition, due to the absence of specific regulations on *B*. *cereus* in food, there is no National Reference Center or National Reference Laboratory to systematically relate the food causing poisonings to the infected patients.”

In light of the concerns above, the *PLOS ONE* editors are issuing this Expression of Concern to make readers aware that the data presented and analyzed in [1] do not appear to be sufficient to support a direct causal relationship between Bt presence and symptomatology or clinical infection.
