# Supplementary material for: Comparative phenotypic, genotypic and genomic analyses of Bacillus thuringiensis associated with foodborne outbreaks in France
Source: PLoS One. 2021 Feb 19;16(2):e0246885. doi: 10.1371/journal.pone.0246885 (PMC7895547; doi:10.1371/journal.pone.0246885)
Supplement: S1 Table — (A) List of the primers used for the study. (B) Conditions of respective PCR amplifications. (PDF) [file pone.0246885.s001.pdf]

A

Gene/PCR	Primer(s)	5'-3' sequence	Expected size (bp)	Reference
-	*M13*	GAGGGTGGCGGCTCT	-	(Guinebretiere and Nguyen-The, 2003)
cytK1	*cytK1F*<br>*cytK1R*	CAATTCCAGGGGCAAGTGTC<br>CCTCGTGCATCTGTTTCATGAG	426	(Guinebretiere et al., 2006)
cytK2	*cytK-2F*<br>*cytK-2R*	CAATCCCTGGCGCTAGTGCA<br>GTGTAGCCTGGACGAAGTTGG	585	(Guinebretiere et al., 2006)
ces	*cesF1*<br>*cesR2*	GGTGACACATTATCATATAAGGTG<br>GTAAGCGAACCTGTCTGTAACAACA	1271	(Ehling-Schulz et al., 2005)
hlyII	*hlyIIF*<br>*hlyIIR*	GATTCTAAAGGAACTGTAG<br>GGTTATCAAGAGTAACTTG	868	(Cadot et al., 2010)
nheA	*45C1*<br>*45C2*	GAGGGGCAAACAGAAGTGAA<br>TGCGAACTTTTGATGATTCG	186	(Moravek et al., 2004)
nheB	*39b1*<br>*39b2*	CCGCTTCTGCAAAATCAAAT<br>TGCGCAGTTGTAACTTGTCC	281	(Moravek et al., 2004)
nheC	*FNheC*<br>*R2NheC*	ACATCCTTTTGCAGCAGAAC<br>CCACCAGCAATGACCATAC	618	(Ngamwongsatit et al., 2008)
hblA	*HA-F1*<br>*HA-R1*	ATTAATACAGGGGATGGAGAAACTT<br>TGATCCTAATACTTCTTCTAGACGCTT	237	(Yang et al., 2005)
hblC	*L2aF*<br>*L2aR*	CGAAAATTAGGTGCGCAATC<br>TAATATGCCTTGCGCAGTTG	411	(Moravek et al., 2004)
hblD	*L1aF*<br>*L1aR*	AGGTCAACAGGCAACGATTC<br>CGAGAGTCCACCAACAACAG	205	(Moravek et al., 2004)
panC	*panCF*<br>*panCR*	TYGGTTTTGTYCCAACRATGG<br>CATAATCTACAGTGCCTTTCG	650	(Guinebretiere et al., 2008)

B

Gene/PCR	*cytK1/2*	*ces*	*hlyII*	*nheA/B/C*	*hblA/C/D*	*panC*
DNA template	10 ng	10 ng	10 ng	10 ng	10 ng	33 ng
PCR buffer	1 x	1 x	1 x	1 x	1 x	1 x
MgCl2	3 mM	3 mM	2 mM	2 mM	2 mM	2.5 mM
dNTPs	0.3 µM	0.5 µM	0.2 µM	0.2 µM	0.2 µM	0.2 µM
Primer(s)	0.5 µM	0.5 µM	0.4 µM	0.4 µM	0,8 µM	0.4 µM
FastStartTaq DNA polymerase (Roche)	1 U	1 U	1 U	1 U	1 U	1 U
Total volume	25 µL	25 µL	25 µL	25 µL	25 µL	100 µL
Positive control	NVH-391-98 and 07CEB02BAC	07CEB021BAC	07CEB02BAC	ATCC 14579	ATCC 14579	-
Negative control	water	water	water	water	water	water
Denaturation	94°C, 5min	95°C, 5min	94°C, 5min	94°C, 5min	94°C, 5min	94°C, 5min
Cycles	30x (94°C for 15s, 58°C for 30s and 72°C for 40s)	30x (95°C for 30s, 58°C for 60s and 72°C for 90s)	30x (95°C for 30s, 58°C for 60s and 72°C for 90s)	30x (94°C for 30s, 53°C for 40s and 72°C for 90s)	30x (94°C for 30s, 53°C for 40s and 72°C for 90s)	30x (94°C for 30s, 55°C for 40s and 72°C for 60s)
Final extenion	72°C for 7 min	72°C for 7 min	72°C for 7 min	72°C for 7 min	72°C for 7 min	72°C for 7 min

Gene/PCR	M13
DNA template	10 ng
PCR buffer	1 x
MgCl2	4 mM
dNTPs	0.9 mM
Primer(s)	2 µM
ReddiamondTaq DNA polymerase (Eurogentec)	2.5 U
Total volume	25 µL
Positive control	ATCC 14579
Negative control	water
Denaturation	94°C, 3min
Cycles	35x (94°C for 1 min, 40°C for 1 min and 68°C for 8 min)
Final extenion	68°C for 8 min
