# Supplementary material for: Comparative phenotypic, genotypic and genomic analyses of Bacillus thuringiensis associated with foodborne outbreaks in France
Source: PLoS One. 2021 Feb 19;16(2):e0246885. doi: 10.1371/journal.pone.0246885 (PMC7895547; doi:10.1371/journal.pone.0246885)
Supplement: S2 Table — +/- = detection/no detection of corresponding activity or gene. The phylogenetic groups were assigned according to the partial sequencing of panC [9]. The attribution to M13 groups (named 1 to 3) was established in this study based on similarity Dice coefficients calculated with Bionumerics. (PDF) [file pone.0246885.s002.pdf]

FBO Bt												
FBO	Isolate	Lecithinase activity	Hemolytic activity	Starch hydrolysis	Phylogenetic group	*cytK1*	*cytK2*	*ces*	*hlyII*	*nhe* A/B/C	*hbl* C/D/A	M13 pattern
1	07CEB29BAC	slight	+	+	IV	-	+	-	-	+	+	3
2	08CEB037BAC	+	slight	+	IV	-	+	-	-	+	+	4
3	08CEB074BAC	+	+	+	IV	-	+	-	-	+	+	1
4	08CEB089BAC	+	+	+	IV	-	+	-	-	+	+	1
5	08CEB121BAC	+	slight	+	IV	-	+	-	-	+	+	1
	08CEB124BAC	+	+	+	IV	-	+	-	-	+	+	1
6	08CEB128BAC	+	+	+	II	-	-	-	+	+	-	x
7	08CEB135BAC	+	+	+	IV	-	+	-	-	+	+	1
	08CEB138BAC	+	+	+	IV	-	+	-	-	+	+	1
8	08CEB145BAC	+	+	+	IV	-	+	-	-	+	+	3
9	09CEB68BAC	+	+	+	IV	-	+	-	-	+	+	3
10	10CEB01BAC	slight	+	+	IV	-	+	-	-	+	+	1
11	10CEB46BAC	+	+	+	IV	-	+	-	-	+	+	1
12	11CEB48BAC	+	+	+	IV	-	+	-	-	+	+	1
13	12CEB17BAC	+	slight	+	IV	-	+	-	-	+	+	3
14	14SBCL08	-	slight	+	IV	-	+	-	-	+	+	1
15	14SBCL16	+	slight	+	IV	-	+	-	-	+	+	3
	14SBCL18	+	slight	+	IV	-	+	-	-	+	+	3
16	14SBCL20	+	slight	+	IV	-	+	-	-	+	+	4
17	14SBCL22	+	slight	+	IV	-	+	-	-	+	+	3
18	14SBCL49	+	+	+	IV	-	+	-	-	+	+	4
19	14SBCL176	+	slight	+	IV	-	+	-	-	+	+	1
20	14SBCL262	+	slight	+	IV	-	+	-	-	+	+	1
21	14SBCL309	+	slight	+	IV	-	+	-	-	+	+	1
22	14SBCL361	+	slight	+	IV	-	+	-	-	+	+	4
	14SBCL362	+	slight	+	IV	-	+	-	-	+	+	4
	14SBCL364	+	slight	+	IV	-	+	-	-	+	+	1
23	14SBCL370	+	slight	+	IV	-	+	-	-	+	+	1
24	14SBCL388	+	slight	+	IV	-	+	-	-	+	+	4
25	15SBCL93	+	slight	+	IV	-	+	-	-	+	+	4
26	15SBCL482	+	slight	+	IV	-	+	-	-	+	+	3
27	15SBCL598	+	slight	+	IV	-	+	-	-	+	+	3
	15SBCL603	+	+	+	IV	-	+	-	-	+	+	3
28	15SBCL915	+	+	+	IV	-	+	-	-	+	+	3
29	15SBCL1007	+	slight	+	IV	-	+	-	-	+	+	4
30	15SBCL1331	+	slight	+	IV	-	+	-	-	+	+	4
31	16 SBCL 350	+	slight	+	IV	-	+	-	-	+	+	3
32	16 SBCL 372	+	slight	+	IV	-	+	-	-	+	+	4
33	16 SBCL 379	+	slight	+	IV	-	+	-	-	+	+	4
	16 SBCL 381	+	+	+	IV	-	+	-	-	+	+	1
34	16 SBCL 417	+	slight	+	IV	-	+	-	-	+	+	4
35	16 SBCL 440	+	slight	+	IV	-	+	-	-	+	+	4
36	16 SBCL 670	+	slight	+	IV	-	+	-	-	+	+	3
	16 SBCL898	+	slight	+	IV	-	+	-	-	+	+	4
37	16 SBCL 1122	+	+	+	IV	-	+	-	-	+	+	4
38	16SBCL1310	+	+	+	IV	-	+	-	-	+	+	3
39	16SBCL1549	+	+	+	IV	-	+	-	-	+	+	3
40	16SBCL1643	+	+	+	IV	-	+	-	-	+	+	1
41	17SBCL 01	+	+	+	IV	-	+	-	-	+	+	4
42	17SBCL263	+	+	+	IV	-	+	-	-	+	+	1
43	17SBCL 334	+	+	+	IV	-	+	-	-	+	+	3
44	17SBCL 429	+	+	+	IV	-	-	-	+	+	+	5
	17SBCL 430	+	+	+	IV	-	+	-	-	+	+	3
45	17SBCL527	+	+	+	IV	-	+	-	-	+	+	4
	17SBCL529	+	+	+	IV	-	+	-	-	+	+	4
46	17SBCL619	+	+	+	IV	-	+	-	-	+	+	4
47	17SBCL885	+	+	+	IV	-	+	-	-	+	+	4
48	17SBCL967	+	+	+	IV	-	+	-	-	+	+	1
49	17SBCL1202	+	+	+	IV	-	+	-	-	+	+	4
Commercial Bt												
Strain	Isolate	Lecithinase activity	Hemolytic activity	Starch hydrolysis	Phylogenetic group	*cytK1*	*cytK2*	*ces*	*hlyII*	*nhe* A/B/C	*hbl* C/D/A	M13 pattern
ABTS-1857	18SBCL209	+	+	+	IV	-	+	-	-	+	+	1
ABTS-1857	18SBCL210	+	+	+	IV	-	+	-	-	+	+	1
ABTS-351	18SBCL212	+	+	+	IV	-	+	-	-	+	+	3
ABTS-351	18SBCL214	+	+	+	IV	-	+	-	-	+	+	3
PB-54	18SBCL215	+	+	+	IV	-	+	-	-	+	+	3
PB-54	18SBCL216	+	+	+	IV	-	+	-	-	+	+	3
SA-11	18SBCL217	+	+	+	IV	-	+	-	-	+	+	3
SA-11	18SBCL218	+	+	+	IV	-	+	-	-	+	+	3
SA-11	18SBCL219	+	+	+	IV	-	+	-	-	+	+	3
EG2348	18SBCL421	+	+	+	IV	-	+	-	-	+	+	3
ABTS-351	18SBCL448	+	+	+	IV	-	+	-	-	+	+	4
ABTS-1857	18SBCL449	+	+	+	IV	-	+	-	-	+	+	1
ABTS-351	18SBCL450	+	+	+	IV	-	+	-	-	+	+	4
BMP144	18SBCL483	+	+	+	IV	-	+	-	+	+	+	2
AM65-52	18SBCL484	+	+	+	IV	-	+	-	+	+	+	2
NB-176	18SBCL485	-	+	+	IV	-	-	-	+	+	+	5
SA-11	18SBCL487	+	+	+	IV	-	+	-	-	+	+	4
SA-12	18SBCL614	+	+	+	IV	-	+	-	-	+	+	3
GC-91	18SBCL617	+	+	+	IV	-	+	-	-	+	+	1
