# Supplementary material for: Comparative phenotypic, genotypic and genomic analyses of Bacillus thuringiensis associated with foodborne outbreaks in France
Source: PLoS One. 2021 Feb 19;16(2):e0246885. doi: 10.1371/journal.pone.0246885 (PMC7895547; doi:10.1371/journal.pone.0246885)
Supplement: S3 Table — * The Nhe absorbance corresponds to the coloration intensity, obtained with the BDE VIATM kit (3M-Tecra), according to the manufacturer’s recommendations. In the tested conditions, the limit of detection corresponds to Nhe Absorbance (414nm) = 0.2. ** The Hbl dilution values correspond to the highest dilution for which Hbl remained detectable, using the immunoenzymatic kit BCET-RPLA (Oxoïd), and according to the manufacturer’s recommendations. nd = not detected. Dilution = 1 is the limit of detection of Hbl and corresponds to a detection of 2ng/ml of Hbl components. (PDF) [file pone.0246885.s003.pdf]

FBO-Bt		
Strain ID	Nhe absorbance*	Hbl dilution**
08CEB037BAC	0.527	1/32
08CEB121BAC	0.209	1/64
08CEB124BAC	0.308	1/64
10CEB01BAC	0.241	1/64
10CEB46BAC	0.233	1/16
14SBCL08	0.405	1/32
14SBCL16	0.590	1/64
14SBCL18	0.590	1/64
14SBCL20	0.901	1/64
14SBCL22	0.564	1/64
14SBCL176	0.432	1/32
14SBCL262	0.441	1/32
14SBCL370	0.330	1/32
16SBCL417	0.983	1/64
16SBCL440	0.719	1/64
16SBCL1310	0.677	1/64
17SBCL334	0.255	1/64
17SBCL527	0.26	1/64
17SBCL529	0.425	1/64
17SBCL619	0.268	1/16
17SBCL967	0.287	1/64

Commercial Bt		
Strain ID	Nhe absorbance*	Hbl dilution**
18SBCL216A	0.743	1/64
18SBCL421A	0.266	1/64
18SBCL448A	0.411	1/64
18SBCL449A	0.233	1/64
18SBCL483A	0.531	nd
18SBCL484A	0.619	1/16
18SBCL485A	0.294	1/64
18SBCL487A	0.403	1/64
18SBCL614A	0.407	1/16
18SBCL617A	0.339	1/16
