# Supplementary material for: Comparative phenotypic, genotypic and genomic analyses of Bacillus thuringiensis associated with foodborne outbreaks in France
Source: PLoS One. 2021 Feb 19;16(2):e0246885. doi: 10.1371/journal.pone.0246885 (PMC7895547; doi:10.1371/journal.pone.0246885)
Supplement: S4 Table — a the attribution to Bacillus thuringiensis (Bt) species was determined by detection of parasporal crystals by phase-contrast microscopy. b the phylogenetic clustering was based on panC sequences similarities [9]. c All the sequencing data obtained from this study are associated with the BioProject PRJNA547495. d,e The strains used for genome assemblies and the SNP calling analysis are indicated with a “x” in the columns “reference assemblies” and “iVARCall2”, respectively. (PDF) [file pone.0246885.s004.pdf]

Isolate/strain	Bt/non Bt[a]	panC group [b]	Source	Origin	Accession[c]	Reference assemblies[d]	iVARCall2 [e]
07CEB29	Bt	IV	this study	FBO	SAMN12147181		x
07CEB30	Bt	IV	this study	FBO	SAMN12147182		x
07CEB31	Bt	IV	this study	FBO	SAMN12147183		x
07CEB32	Bt	IV	this study	FBO	SAMN12147184		x
08CEB121	Bt	IV	this study	FBO	SAMN12147185		x
08CEB122	Bt	IV	this study	FBO	SAMN12147186		x
08CEB123	Bt	IV	this study	FBO	SAMN12147187		x
08CEB124	Bt	IV	this study	FBO	SAMN12147188		x
08CEB125	Bt	IV	this study	FBO	SAMN12147189		x
08CEB126	Bt	IV	this study	FBO	SAMN12147190		x
08CEB127	Bt	IV	this study	FBO	SAMN12147191		x
08CEB128	Bt	II	this study	FBO	SAMN12217386		
08CEB133	non Bt	IV	this study	FBO	SAMN12147192		x
08CEB135	Bt	IV	this study	FBO	SAMN12147193		x
08CEB138	Bt	IV	this study	FBO	SAMN12147194		x
08CEB145	Bt	IV	this study	FBO	SAMN12147195		x
08CEB037	Bt	IV	this study	FBO	SAMN12147196		x
08CEB38	Bt	IV	this study	FBO	SAMN12147197		x
08CEB69	non Bt	VI	this study	FBO	SAMN12217387		
08CEB71	non Bt	VI	this study	FBO	SAMN12217388		
08CEB72	non Bt	VI	this study	FBO	SAMN12217389		
08CEB74	Bt	IV	this study	FBO	SAMN12147198		x
08CEB78	non Bt	IV	this study	FBO	SAMN12147199		x
08CEB89	Bt	IV	this study	FBO	SAMN12147200		x
09CEB18	non Bt	IV	this study	FBO	SAMN12147201		x
09CEB68	Bt	IV	this study	FBO	SAMN12147202		x
09CEB69	Bt	IV	this study	FBO	SAMN12147203		x
10CEB01	Bt	IV	this study	FBO	SAMN12147204		x
10CEB02	Bt	IV	this study	FBO	SAMN12147205		x
10CEB03	Bt	IV	this study	FBO	SAMN12147206		x
10CEB04	Bt	IV	this study	FBO	SAMN12147207		x
10CEB05	Bt	IV	this study	FBO	SAMN12147208		x
10CEB29	non Bt	II	this study	FBO	SAMN12217390		
10CEB32	non Bt	IV	this study	FBO	SAMN12147209		x
10CEB46	Bt	IV	this study	FBO	SAMN12147210		x
10CEB47	Bt	IV	this study	FBO	SAMN12147211		x
10CEB48	Bt	IV	this study	FBO	SAMN12147212		x
10CEB49	Bt	IV	this study	FBO	SAMN12147213		x
10CEB50	Bt	IV	this study	FBO	SAMN12147214		x
10CEB51	Bt	IV	this study	FBO	SAMN12147215		x
10CEB81	non Bt	III	this study	FBO	SAMN12217391		
11CEB48	Bt	IV	this study	FBO	SAMN12147216		x
11CEB61	non Bt	II	this study	FBO	SAMN12217392		
12CEB14	non Bt	III	this study	FBO	SAMN12217393		
12CEB17	Bt	IV	this study	FBO	SAMN12147217		x
14SBCL08	Bt	IV	this study	FBO	SAMN12147218		x
14SBCL108	non Bt	II	this study	FBO	SAMN12217394		
14SBCL16	Bt	IV	this study	FBO	SAMN12147219		x
14SBCL49	Bt	IV	this study	FBO	SAMN12147220		x
14SBCL176	Bt	IV	this study	FBO	SAMN12147221		x
14SBCL177	Bt	IV	this study	FBO	SAMN12147222		x
14SBCL178	Bt	IV	this study	FBO	SAMN12147223		x
14SBCL179	Bt	IV	this study	FBO	SAMN12147224		x
14SBCL18	Bt	IV	this study	FBO	SAMN12147225		x
14SBCL180	Bt	IV	this study	FBO	SAMN12147226		x
14SBCL20	Bt	IV	this study	FBO	SAMN12147227		x
14SBCL22	Bt	IV	this study	FBO	SAMN12147228		x
14SBCL262	Bt	IV	this study	FBO	SAMN12147229		x
14SBCL263	Bt	IV	this study	FBO	SAMN12147230		x
14SBCL264	Bt	IV	this study	FBO	SAMN12147231		x
14SBCL265	Bt	IV	this study	FBO	SAMN12147232		x
14SBCL266	Bt	IV	this study	FBO	SAMN12147233		x
14SBCL309	Bt	IV	this study	FBO	SAMN12147234		x
14SBCL310	Bt	IV	this study	FBO	SAMN12147235		x
14SBCL311	Bt	IV	this study	FBO	SAMN12147236		x
14SBCL312	Bt	IV	this study	FBO	SAMN12147237		x
14SBCL313	Bt	IV	this study	FBO	SAMN12147238		x
14SBCL359	non Bt	IV	this study	FBO	SAMN12147239		x
14SBCL361	Bt	IV	this study	FBO	SAMN12147240		x
14SBCL362	Bt	IV	this study	FBO	SAMN12147241		x
14SBCL364	Bt	IV	this study	FBO	SAMN12147242		x
14SBCL370	Bt	IV	this study	FBO	SAMN12147243		x
14SBCL371	Bt	IV	this study	FBO	SAMN12147244		x
14SBCL372	Bt	IV	this study	FBO	SAMN12147245		x
14SBCL373	Bt	IV	this study	FBO	SAMN12147246		x

14SBCL374	Bt	IV	this study	FBO	SAMN12147247	x
14SBCL388	Bt	IV	this study	FBO	SAMN12147248	x
15SBCL1007	Bt	IV	this study	FBO	SAMN12147249	x
15SBCL1008	Bt	IV	this study	FBO	SAMN12147250	x
15SBCL1009	Bt	IV	this study	FBO	SAMN12147251	x
15SBCL1010	Bt	IV	this study	FBO	SAMN12147252	x
15SBCL1011	Bt	IV	this study	FBO	SAMN12147253	x
15SBCL1103	non Bt	IV	this study	FBO	SAMN12147254	x
15SBCL1331	Bt	IV	this study	FBO	SAMN12147255	x
15SBCL439	non Bt	III	this study	FBO	SAMN12217395	
15SBCL482	Bt	IV	this study	FBO	SAMN12147256	x
15SBCL598	Bt	IV	this study	FBO	SAMN12147257	x
15SBCL599	Bt	IV	this study	FBO	SAMN12147258	x
15SBCL600	Bt	IV	this study	FBO	SAMN12147259	x
15SBCL601	Bt	IV	this study	FBO	SAMN12147260	x
15SBCL602	Bt	IV	this study	FBO	SAMN12147261	x
15SBCL603	Bt	IV	this study	FBO	SAMN12147262	x
15SBCL605	Bt	IV	this study	FBO	SAMN12147263	x
15SBCL606	Bt	IV	this study	FBO	SAMN12147264	x
15SBCL607	Bt	IV	this study	FBO	SAMN12147265	x
15SBCL85	non Bt	IV	this study	FBO	SAMN12147266	x
15SBCL86	non Bt	IV	this study	FBO	SAMN12147267	x
15SBCL88	non Bt	III	this study	FBO	SAMN12217396	
15SBCL914	non Bt	III	this study	FBO	SAMN12217397	
15SBCL915	Bt	IV	this study	FBO	SAMN12147268	x
15SBCL93	Bt	IV	this study	FBO	SAMN12147269	x
16SBCL1121	non Bt	III	this study	FBO	SAMN12217398	
16SBCL1122	Bt	IV	this study	FBO	SAMN12147270	x
16SBCL1267	non Bt	II	this study	FBO	SAMN12217399	
16SBCL1310	Bt	IV	this study	FBO	SAMN12147271	x
16SBCL1549	Bt	IV	this study	FBO	SAMN12147272	x
16SBCL1643	Bt	IV	this study	FBO	SAMN12147273	x
16SBCL169	non Bt	IV	this study	FBO	SAMN12147274	x
16SBCL350	Bt	IV	this study	FBO	SAMN12147275	x
16SBCL372	Bt	IV	this study	FBO	SAMN12147276	x
16SBCL379	Bt	IV	this study	FBO	SAMN12147277	x
16SBCL380	Bt	IV	this study	FBO	SAMN12147278	x
16SBCL381	Bt	IV	this study	FBO	SAMN12147279	x
16SBCL404	non Bt	IV	this study	FBO	SAMN12147280	x
16SBCL417	Bt	IV	this study	FBO	SAMN12147281	x
16SBCL418	Bt	IV	this study	FBO	SAMN12147282	x
16SBCL440	Bt	IV	this study	FBO	SAMN12147283	x
16SBCL646	non Bt	V	this study	FBO	SAMN12217400	
16SBCL670	Bt	IV	this study	FBO	SAMN12147284	x
16SBCL898	Bt	IV	this study	FBO	SAMN12147285	x
17SBCL01	Bt	IV	this study	FBO	SAMN12147286	x
17SBCL1086	non Bt	IV	this study	FBO	SAMN12147287	x
17SBCL1202	Bt	IV	this study	FBO	SAMN12147288	x
17SBCL1228	non Bt	II	this study	FBO	SAMN12217401	
17SBCL263	Bt	IV	this study	FBO	SAMN12147289	x
17SBCL264	Bt	IV	this study	FBO	SAMN12147290	x
17SBCL265	Bt	IV	this study	FBO	SAMN12147291	x
17SBCL266	Bt	IV	this study	FBO	SAMN12147292	x
17SBCL267	Bt	IV	this study	FBO	SAMN12147293	x
17SBCL268	Bt	IV	this study	FBO	SAMN12147294	x
17SBCL273	non Bt	VI	this study	FBO	SAMN12217402	
17SBCL334	Bt	IV	this study	FBO	SAMN12147295	x
17SBCL429	Bt	IV	this study	FBO	SAMN12147296	x
17SBCL430	Bt	IV	this study	FBO	SAMN12147297	x
17SBCL527	Bt	IV	this study	FBO	SAMN12147298	x
17SBCL528	Bt	IV	this study	FBO	SAMN12147299	x
17SBCL529	Bt	IV	this study	FBO	SAMN12147300	x
17SBCL530	Bt	IV	this study	FBO	SAMN12147301	x
17SBCL531	Bt	IV	this study	FBO	SAMN12147302	x
17SBCL619	Bt	IV	this study	FBO	SAMN12147303	x
17SBCL620	Bt	IV	this study	FBO	SAMN12147304	x
17SBCL621	Bt	IV	this study	FBO	SAMN12147305	x
17SBCL622	Bt	IV	this study	FBO	SAMN12147306	x
17SBCL623	Bt	IV	this study	FBO	SAMN12147307	x
17SBCL879	non Bt	II	this study	FBO	SAMN12217403	
17SBCL885	Bt	IV	this study	FBO	SAMN12147308	x
17SBCL908	non Bt	IV	this study	FBO	SAMN12147309	x
17SBCL967	Bt	IV	this study	FBO	SAMN12147310	x
17SBCL968	Bt	IV	this study	FBO	SAMN12147311	x
17SBCL969	Bt	IV	this study	FBO	SAMN12147312	x
17SBCL970	Bt	IV	this study	FBO	SAMN12147313	x
17SBCL971	Bt	IV	this study	FBO	SAMN12147314	x

18SBCL209	Bt	IV	this study	pesticide	SAMN12147315		x
18SBCL210	Bt	IV	this study	pesticide	SAMN12147316		x
18SBCL212	Bt	IV	this study	pesticide	SAMN12147317		x
18SBCL214	Bt	IV	this study	pesticide	SAMN12147318		x
18SBCL215	Bt	IV	this study	pesticide	SAMN12147319		x
18SBCL216	Bt	IV	this study	pesticide	SAMN12147320		x
18SBCL217	Bt	IV	this study	pesticide	SAMN12147321		x
18SBCL218	Bt	IV	this study	pesticide	SAMN12147322		x
18SBCL219	Bt	IV	this study	pesticide	SAMN12147323		x
18SBCL421	Bt	IV	this study	pesticide	SAMN12147324		x
18SBCL448	Bt	IV	this study	pesticide	SAMN12147325		x
18SBCL449	Bt	IV	this study	pesticide	SAMN12147326		x
18SBCL450	Bt	IV	this study	pesticide	SAMN12147327		x
18SBCL483	Bt	IV	this study	pesticide	SAMN12147328		x
18SBCL484	Bt	IV	this study	pesticide	SAMN12147329		x
18SBCL485	Bt	IV	this study	pesticide	SAMN12147330		x
18SBCL487	Bt	IV	this study	pesticide	SAMN12147331		x
18SBCL614	Bt	IV	this study	pesticide	SAMN12147332		x
18SBCL617	Bt	IV	this study	pesticide	SAMN12147333		x
CIP53137	Bt	IV	this study	"Collection de l'Institut Pasteur"	SAMN12147334		x
Ba_Ames	non Bt	III	NCBI	deadcow, Texas	NC_003997.3		
Bc_AH187	non Bt	III	NCBI	Unknown	CP001177	x	
Bc_ATCC_14579	non Bt	IV	NCBI	Cowshed, American Type Culture Collection	NC_011658.1	x	
Bc_*cytotoxicus*_NVH_391_98	non Bt	VII	NCBI	FBO, France	NC_009674.1	x	
Bc_*mycoides*_DSM_2048	non Bt	VI	NCBI	soil	NZ_CM000742.1	x	
Bc_*pseudomycoides*_DSM_12442	non Bt	I	NCBI	soil, Ghana	NZ_CM000745.1	x	
Bc_*toyonensis*_BCT-7112	non Bt	V	NCBI	derivedstrain, Japan	NC_022781.1	x	
Bc_*weihenstephanensis*_WSBC10204	non Bt	VI	NCBI	pasteurizedmilk	NZ_CP009746.1	x	
Bc_*wiedmannii*_MM3	non Bt	II	NCBI	Unknown	NZ_CM000718.1	x	
Bt_Al_Hakam	Bt	III	NCBI	Unknown	NC_008600.1		
Bt_*allesti*_BGSC4C1	Bt	IV	NCBI	soil, Bombyx mori	NZ_CP015176.1		
Bt_ATCC10792	Bt	IV	NCBI	Unknown, American Type Culture Collection	NZ_CP021061.1		
Bt_Bc601	Bt	IV	NCBI	Unknown	NZ_CP015150.1		
Bt_BMB171	Bt	IV	NCBI	Unknown	NC_014171.1		
Bt_BM_BT15426	Bt	III	NCBI	culture	NZ_CP020723.1		
Bt_Bt18247	Bt	IV	NCBI	Unknown	NZ_CP015250.1		
Bt_Bt185	Bt	IV	NCBI	soil	NZ_CP014282.1		
Bt_C15	Bt	IV	NCBI	soil	NZ_CP021436.1		
Bt_c25	Bt	IV	NCBI	soil	NZ_CP022345.1		
Bt_*chinensis*_CT43	Bt	IV	NCBI	Unknown	NC_017208.1		
Bt_CTC	Bt	II	NCBI	soil	NZ_CP013274.1		
Bt_*finitimus*_YBT020	Bt	III	NCBI	Unknown	NC_017200.1		
Bt_*galleriae*_HD29	Bt	IV	NCBI	Dendrolimussibiricus	NZ_CP010089.1		
Bt_HD1002	Bt	IV	NCBI	Sewage	NZ_CP009351.1		
Bt_HD1011	Bt	III	NCBI	Unknown	NZ_CP009335.1		
Bt_HD12	Bt	IV	NCBI	soil	NZ_CP014847.1		
Bt_HD571	Bt	III	NCBI	Unknown	NZ_CP009600.1		
Bt_HD682	Bt	III	NCBI	Unknown	NZ_CP009720.1		
Bt_HD771	Bt	IV	NCBI	Unknown	NC_018500.1		
Bt_HD789	Bt	IV	NCBI	Unknown	NC_018508.1		
Bt_HS18_1	Bt	IV	NCBI	soil	NZ_CP012099.1		
Bt_*indiana*_HD521	Bt	IV	NCBI	soil	NZ_CP010106.1		
Bt_*israelensis*_AM6552	Bt	IV	NCBI	Unknown	NZ_CP013275.1	x	
Bt_KNU07	Bt	IV	NCBI	ginseng	NZ_CP016588.1		
Bt_*konkukian*_97-27	Bt	III	NCBI	Unknown	NC_005957.1		
Bt_*kurstaki*_HD1	Bt	IV	NCBI	soil	NZ_CP004870.1	x	
Bt_*kurstaki*_HD73	Bt	IV	NCBI	Unknown	NC_020238.1	x	
Bt_*kurstaki*_YBT-1520	Bt	IV	NCBI	soil	NZ_CP004858.1	x	
Bt_L7601	Bt	IV	NCBI	Unknown	NZ_CP020002.1		
Bt_LM1212	Bt	III	NCBI	Oryctes gigas	NZ_CP024771.1		
Bt_MC28	Bt	V	NCBI	Unknown	NC_018693.1		
Bt_*morrisoni*_BGSC4AA1	Bt	IV	NCBI	soil	NZ_CP010577.1		
Bt_MYBT18246	Bt	IV	NCBI	Unknown	NZ_CP015350.1		
Bt_QZL38	Bt	IV	NCBI	soil	NZ_CP032608.1		
Bt_SCG04_02	Bt	IV	NCBI	soil	NZ_CP017577.1		
Bt_*thuringiensis*_IS5056	Bt	IV	NCBI	Unknown	NC_020376.1		
Bt_*tolworthi*	Bt	IV	NCBI	Unknown	NZ_AP014864.1		
Bt_YBT1518	Bt	IV	NCBI	soil	NC_022873.1		
Bt_YC10	Bt	IV	NCBI	tobaccoroots	NZ_CP011349.1		
Bt_YGd22_03	Bt	IV	NCBI	soil	NZ_CP019230.1		
Bt_YWC2-8	Bt	IV	NCBI	soil	NZ_CP013055.1		
4J1	Bt	IV	NCBI	Heliothisassulta	NFDU00000000		
4J3	Bt	IV	NCBI	Plodiainterpunctella	NFDW00000000		
4J5	Bt	IV	NCBI	Plodiainterpunctella	NFDY00000000		

T07005	Bt	IV	NCBI	Unknown	NFEO00000000
T07128	Bt	IV	NCBI	Unknown	NFER00000000
T07151	Bt	IV	NCBI	Unknown	NFET00000000
Leapi01	Bt	IV	NCBI	Unknown	AMXS00000000.2
T07153	Bt	IV	NCBI	Unknown	NFEU00000000
4Q1	Bt	IV	NCBI	Culicidaelarvae	NFED00000000
T03a001	Bt	IV	NCBI	Ephestiakühniella	ACND00000000.1
I13	Bt	IV	NCBI	Unknown	NFEN00000000
