# Supplementary figures and images for: Comparative phenotypic, genotypic and genomic analyses of Bacillus thuringiensis associated with foodborne outbreaks in France
Source: PLoS One. 2021 Feb 19;16(2):e0246885. doi: 10.1371/journal.pone.0246885 (PMC7895547; doi:10.1371/journal.pone.0246885)

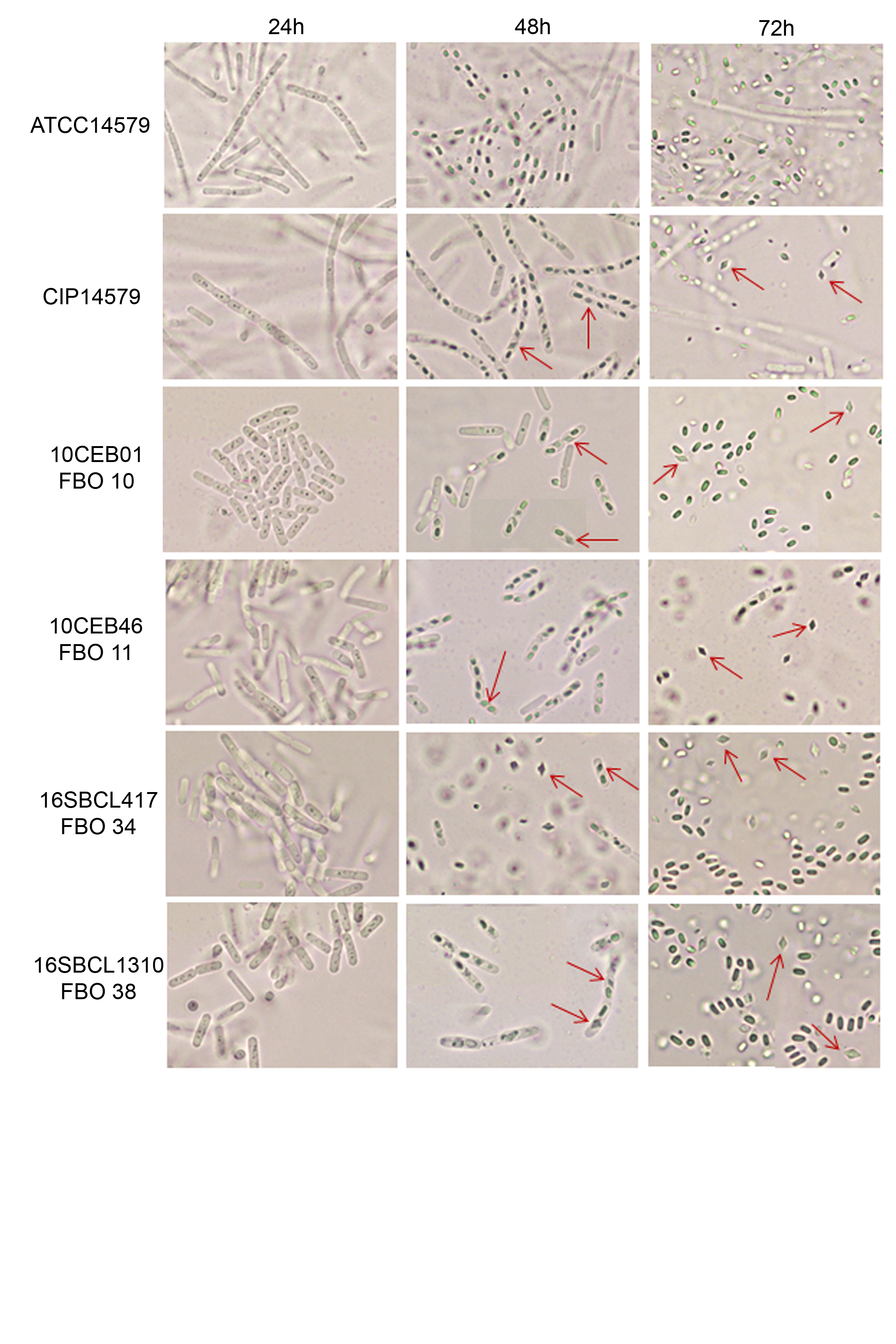

Supplement: S1 Fig — Images were acquired by phase-contrast microscopy. Strains ATCC14579 and CIP53137 were used as negative and positive controls, respectively. Red arrows indicate the presence of crystals. (TIF) [file pone.0246885.s005.tif]

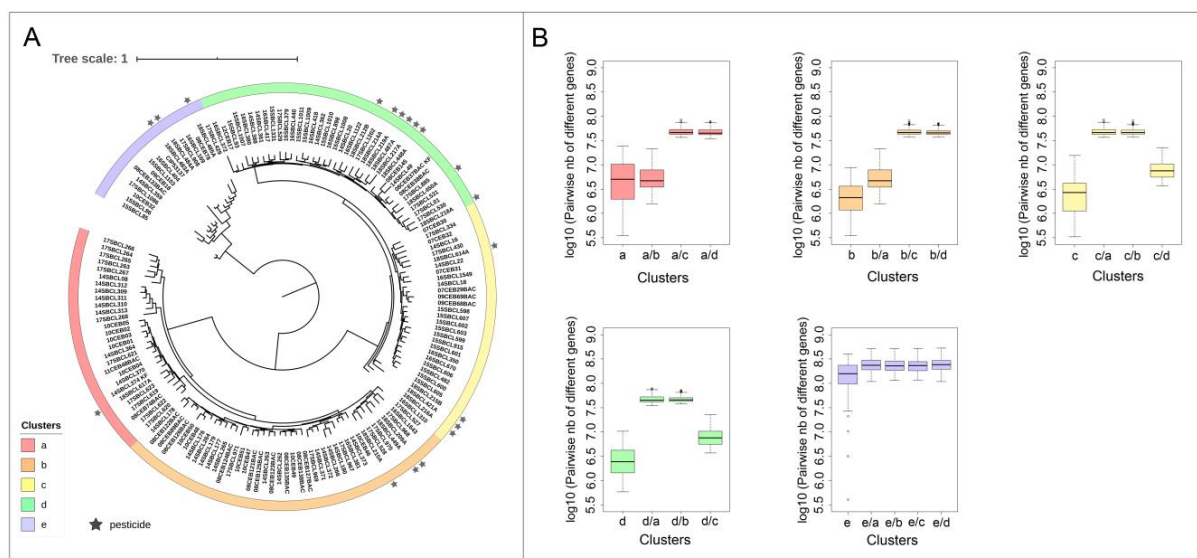

Supplement: S2 Fig — (A) Accessory binary genes tree, established with ROARY [44] and iQtree [43]. The visualization was done using iTOL [46]. (B) Diagrams of the distributions of intra- and inter-clusters log10 (number of different genes). (PDF) [file pone.0246885.s006.pdf]

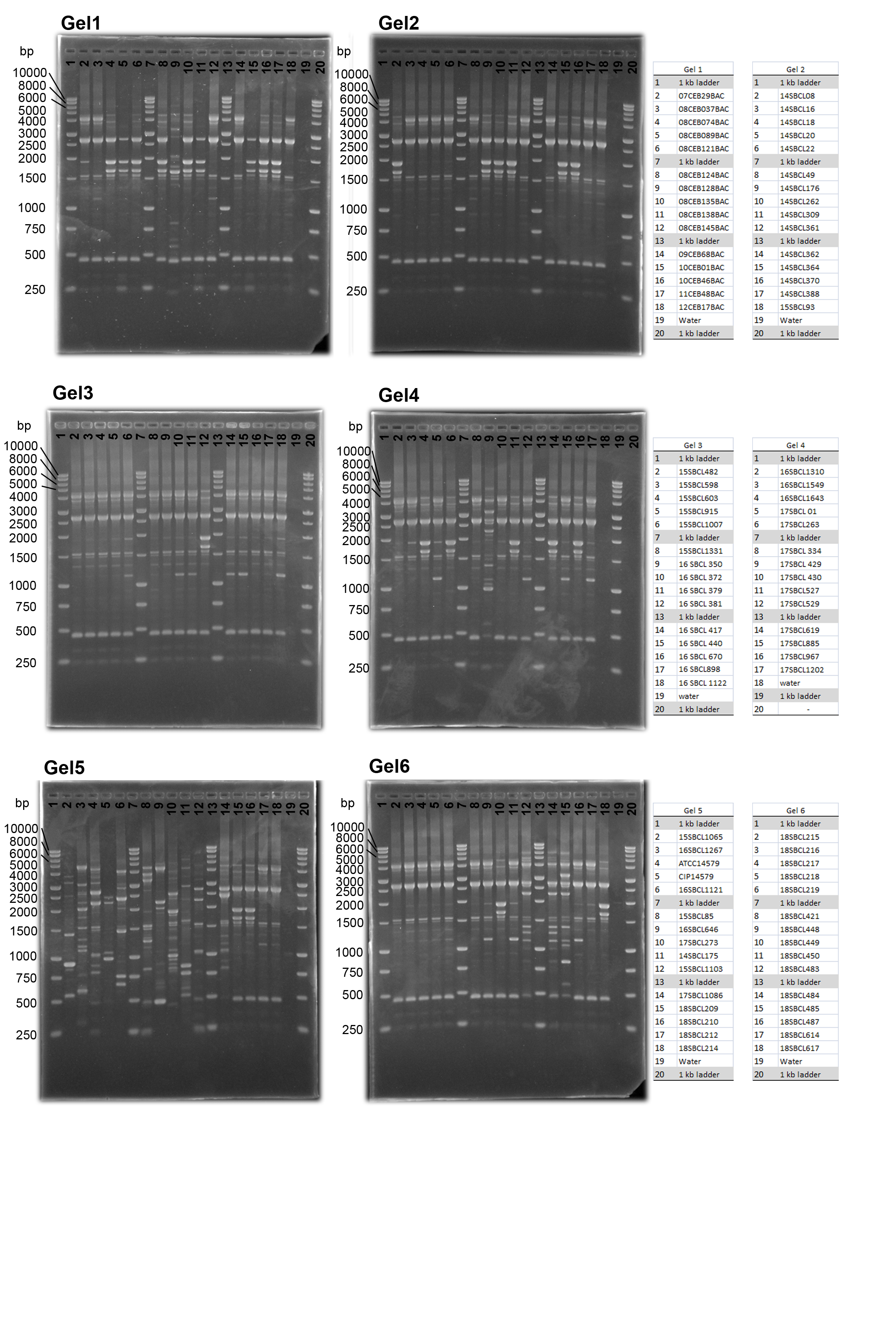

Supplement: S1 Raw images — (TIF) [file pone.0246885.s007.tif]
